# Dopaminergic Gene Polymorphisms and Personality Traits in Athletes: A Systematic Review and Exploratory Quantitative Synthesis

**DOI:** 10.3390/ijms27198484

**Published:** 2026-09-23

**Authors:** Kinga Łosińska, Amr Saber Hamza, Julia Depta, Remigiusz Recław, Anna Grzywacz, Łukasz Jaworski, Adam Maszczyk

**Affiliations:** 1 Department of Physical Education, Gdansk University of Physical Education and Sport, 80-336 Gdańsk, Poland; julia.depta@awf.gda.pl (J.D.); anna.grzywacz@awf.gda.pl (A.G.); a.maszczyk@awf.katowice.pl (A.M.); 2 Faculty of Sports Science, Sohag University, Sohag 82524, Egypt; amr.saber@phyedu.sohag.edu.eg; 3 Department of Public Health, Epidemiology and Behavioral Genetics, Pomeranian Medical University in Szczecin, 70-111 Szczecin, Poland; remigiusz.reclaw@pum.edu.pl; 4 Cardiosurgery Department, Specialized Hospital, 84-200 Wejherowo, Poland; ljaworski75@gmail.com; 5 Institute of Sport Sciences, The Jerzy Kukuczka Academy of Physical Education in Katowice, 40-065 Katowice, Poland

**Keywords:** dopamine, *DRD2*, *DRD4*, *COMT*, *DAT1*, *ANKK1*, novelty seeking, combat sports, personality traits, systematic review

## Abstract

Personality traits relevant to sport performance are partly heritable, and dopaminergic genes have been proposed as contributors to individual differences in athlete personality. However, evidence for these associations remains inconsistent. To systematically review and synthesize evidence on associations between dopaminergic gene polymorphisms and personality traits in athletes. Following PRISMA 2020, STREGA, and SWiM recommendations, databases were systematically searched using predefined eligibility criteria. Fourteen primary association studies met the eligibility criteria; one systematic review was identified and retained as a background source only, contributing no primary data. The protocol was prospectively registered in PROSPERO (CRD420261430605). Study quality was assessed using a STREGA-informed checklist. Standardized mean differences (Hedges’ *g*) were calculated where genotype-specific means and standard deviations were available. Because of overlapping cohorts and heterogeneous reporting, conventional meta-analysis was not feasible; therefore, an exploratory quantitative synthesis, vote counting, and allele/genotype frequency analyses were performed. Most studies involved male Caucasian Polish combat-sport athletes, with two independent cohorts from Egypt and Turkey, and examined *DRD2*, *DRD4*, *COMT*, *DAT1*/*SLC6A3*, and *ANKK1* polymorphisms. The most frequently examined association was between *DRD2* rs1799732 and reward dependence, for which two cohorts reported small, directionally concordant estimates whose confidence intervals both included the null (g = −0.18, 95% CI −0.44 to +0.09; g = −0.20, 95% CI −0.40 to +0.01); because these cohorts share controls and overlapping participants, they were not pooled and do not constitute independent replication. Other associations were generally small and study-specific. The *COMT* rs4680 Met allele was less frequent in athletes than controls (OR 0.75, 95% CI 0.59–0.96). Evidence was limited by overlapping cohorts, small samples, methodological heterogeneity, and predominant reliance on a single research network; the few studies conducted outside that network reported largely null genotype–personality associations. Current evidence is insufficient to establish associations between dopaminergic polymorphisms and sport-related personality traits: the reported associations are derived almost entirely from a single, non-independent research network, and no gene × trait association has been replicated in an independent sample. Certainty of the cumulative evidence, appraised with the Venice criteria for genetic associations, was weak for every gene × trait association examined. Large, independent, multicenter studies using standardized phenotyping, together with polygenic and epigenetic approaches, are needed before any inference can be drawn.

## 1. Introduction

### 1.1. Personality in Sports Performance

Personality traits are relatively stable, biologically anchored dispositions that shape how athletes appraise competition, regulate arousal, tolerate risk, and persist under fatigue [1]. Across sport disciplines, conscientiousness and extraversion are the traits most consistently associated with athletic attainment, whereas neuroticism tends to be lower in athletes than in non-athletes [2,3]. In combat sports, disciplines defined by direct physical confrontation through striking, grappling, or mixed engagement, traits such as novelty seeking, sensation seeking, impulsivity, harm avoidance, and conscientiousness are of particular interest because they map onto behaviors that are functionally consequential in the ring or on the mat [4,5]. Understanding the dispositional profile of combat-sport athletes and its biological correlates is therefore relevant both to talent identification and to individualized psychological support (Table 1).

### 1.2. Neurobiology of Personality

Two influential frameworks connect personality to neurobiology. Cloninger’s psychobiological model links temperament dimensions, novelty seeking, harm avoidance, reward dependence and persistence to specific neurotransmitter systems, proposing that novelty seeking reflects dopaminergic activity, harm avoidance serotonergic activity, and reward dependence noradrenergic activity [6,7]. The Five Factor Model (operationalized by the NEO inventories) describes personality through neuroticism, extraversion, openness, agreeableness, and conscientiousness, several of which show partial convergence with Cloninger’s dimensions [1]. Both models are heritable and provide candidate phenotypes for genetic association studies [7].

### 1.3. The Dopaminergic System

Dopamine is a central neuromodulator of reward anticipation, incentive salience, motor preparation, and cognitive control within mesolimbic and mesocortical circuits [8]. Individual differences in dopaminergic signaling, receptor density and affinity, and synaptic dopamine availability and reuptake plausibly underlie individual differences in approach motivation, reward sensitivity, and impulse control [8,9]. Common functional polymorphisms in dopamine pathway genes have therefore been studied extensively as candidate correlates of approach-related personality traits [10].

### 1.4. Candidate Polymorphisms

Five variants constitute the prespecified analytical core of this review. They were selected a priori in the registered protocol on two grounds: each has documented or strongly inferred functional consequences for dopaminergic signaling at a distinct point in the pathway (receptor density and expression, prefrontal catabolism, synaptic clearance, and the *ANKK1*/*DRD2* locus), and each is among the most extensively studied dopaminergic variants in personality research, so that they offered the greatest prospect of cross-study synthesis. The candidate set was therefore not defined by the evidence that the search subsequently retrieved. Eligibility was defined at the level of the five genes rather than these five variants alone, so that other variants within *DRD2*, *DRD4*, *COMT*, *DAT1*/*SLC6A3*, and *ANKK1* (for example *DRD2* rs1076560, rs1800498, rs6276 and rs1079597) were eligible and are included below. Variants in other dopaminergic genes, such as *DRD1*, *DRD3*, *DRD5*, *DBH*, *TH*, and *MAOA*, were excluded by design; this restriction is acknowledged as a limitation in Section 4.4. The five core variants are as follows:

- *DRD4 exon-III VNTR*: A 48 bp variable number tandem repeat in the third exon; the 7-repeat (long) allele encodes a receptor with attenuated cAMP signaling and has been the most studied personality-related dopaminergic variant, historically linked to novelty seeking [11,12], although later meta-analyses have substantially attenuated this association [10,13].
- *COMT rs4680 (Val158Met)*: A functional substitution altering catechol-O-methyltransferase activity and thus prefrontal dopamine catabolism; the Val/Met balance underlies the “warrior/worrier” account of stress-related cognitive performance [14].
- *DRD2 rs1799732 (−141C Ins/Del)*: A promoter insertion/deletion affecting D2 receptor expression, studied in relation to reward dependence and harm avoidance [8].
- *DAT1/SLC6A3 40-bp VNTR*: A 3′-UTR repeat polymorphism influencing dopamine transporter expression and synaptic dopamine clearance, studied in relation to impulsivity and sensation seeking [5,15].
- *ANKK1 rs1800497 (Taq1A)*: Located in the *ANKK1* gene adjacent to *DRD2*; the A1 allele is associated with reduced striatal D2 receptor density and with reward-related and sensation seeking phenotypes [8,9].

### 1.5. Inconsistency in Athlete Studies

Despite this conceptual coherence, the athlete literature is inconsistent, for several reasons. Measurement heterogeneity is pervasive: studies use different instruments (TCI/TCI-R, NEO-FFI, STAI, BIS-11) that operationalize overlapping but non-identical constructs [1,7]. Genetic model heterogeneity is common, with studies comparing genotypes under dominant, recessive, additive, or carrier contrasts. Sample heterogeneity is marked, spanning the sport type, competitive level, sex, and ancestry [4]. Finally, most studies are statistically underpowered for the small effects that candidate-gene personality associations typically produce, and many do not report the descriptive statistics needed for cross-study quantitative synthesis [10] (Table 2).

### 1.6. Rationale for the Present Review

Standardizing effects via the standardized mean difference (Hedges’ *g*) is, in principle, the appropriate route to synthesizing findings across heterogeneous instruments under a harmonized allele-carrier-versus-non-carrier model [29]. This review was undertaken to determine (i) which dopaminergic variants have been studied against validated personality measures in athletes, (ii) the direction and magnitude of associations, expressed as standardized mean differences wherever the source tables permit, with emphasis on combat-sport athletes, and (iii) whether the existing evidence base is sufficient, in independence and in reporting quality, to support a pooled meta-analytic estimate. The answers to these questions are reported in Section 3.4, Section 3.5 and Section 3.6.

## 2. Methods

### 2.1. Protocol and Reporting Standards

The review was conducted and reported in accordance with the PRISMA 2020 statement and, for the genetic association component, with the STREGA reporting extension. Because conventional quantitative pooling proved infeasible, the synthesis is additionally reported in accordance with the Synthesis Without Meta-analysis (SWiM) guidance. The review protocol was prospectively registered in the International Prospective Register of Systematic Reviews (PROSPERO; registration number CRD420261430605, registered 15 January 2026); the full protocol is available from the corresponding author on request. A completed PRISMA 2020 checklist is provided as Supplementary File S1, and the completed SWiM reporting checklist as Supplementary File S3.

Two deviations from the registered protocol are reported. First, the protocol specified a random-effects meta-analysis of gene × trait associations; because no gene × trait cell contained two independent, non-overlapping samples (Section 2.7), the planned pooling was replaced by study-level standardized mean differences, vote counting reported under SWiM guidance, and a single, explicitly labeled transparency estimate. Second, the protocol permitted the supplementary screening of records identified from reference lists and related review articles; no records were identified or screened in this way, and the present review is therefore based on database searching alone.

### 2.2. Eligibility Criteria

**Inclusion:** (i) empirical, peer reviewed studies with original data; (ii) human participants from combat sport or broader athletic populations; (iii) assessment of at least one core dopaminergic polymorphism (*DRD2*, *DRD4*, *COMT*, *DAT1*/*SLC6A3*, *ANKK1*); (iv) at least one personality outcome measured with a validated instrument (TCI/TCI-R, NEO-FFI, STAI, BIS-11, or equivalent); and (v) a comparison structure permitting genotype, phenotype, or athlete–control contrast.

**Exclusion:** reviews, editorials, letters, and conference abstracts (retained only as background, not as primary data); studies without a validated personality outcome; studies reporting only genotype frequencies with no personality measure; and animal or non-original reports. Review articles were retained as background sources only: they were not counted among the included studies and contributed no primary data to the synthesis. The PICOS framework is summarized in Table 3.

### 2.3. Information Sources and Search Strategy

Five electronic databases were searched: PubMed, Scopus, Web of Science Core Collection, SPORTDiscus, and Embase. Each database was searched from inception to 15 March 2026, the date of the last search. In all databases, the search was limited to human studies, peer-reviewed original reports, and studies employing a validated personality or temperament instrument. The search combined three concept blocks with the Boolean operator AND, dopaminergic genes, personality and temperament constructs, and athletic or combat-sport populations, as summarized in Table 4. The complete strategy as executed in each database, including field tags, truncation, and applied limits, is reproduced in Supplementary Table S1, together with the date on which each search was run and the number of records it returned. No registers, trial registries, preprint servers, websites, or organizational sources were searched, no citation searching was undertaken, and no authors were contacted for additional records. The database searches returned 1245 records in total (PubMed, 312; Scopus, 398; Web of Science Core Collection, 287; SPORTDiscus, 156; Embase, 92), from which 245 duplicate records were removed via automated deduplication in EndNote 20 (Clarivate, Philadelphia, PA, USA) followed by the manual verification of uncertain cases; 1000 records entered title and abstract screening.

### 2.4. Screening and Study Selection Process

Records were screened against six prespecified eligibility domains: appropriate study design; human participants; athletic or combat-sport population; analysis of at least one dopaminergic polymorphism; a reported genotype personality association; and use of a validated personality instrument. Each record was rated on every domain as yes, maybe, or no at the title and abstract stage. Screening was supported by an automated, large language model-based screening tool (Elicit; Elicit Research, PBC, Oakland, CA, USA; https://elicit.com, accessed on 17 September 2026), which applied the six domains to the title and abstract of every record and returned criterion-level ratings together with an overall screening score; records meeting all six domains were retained for full-text retrieval. Two reviewers (K.Ł. and J.D.) verified the automated screening decisions independently: all retained records and a random 10% sample of the excluded records were checked against the eligibility criteria, and disagreements were resolved via consensus, with a third reviewer (A.M.) consulted where agreement could not be reached. During revision, this verification was extended because the automated screening tool had operationalized the population domain more narrowly than the eligibility criteria, which admit combat sport or broader athletic populations: records describing athletes without specifying the discipline had been rated as uncertain on that domain and consequently fell below the retention threshold. Every excluded record that had been rated yes or maybe on all five non-population domains (human participants, study design, dopaminergic variant, genotype personality association, and validated instrument) and whose title or abstract referred to athletes, sport, or training was therefore reexamined by two reviewers (K.Ł. and J.D.), irrespective of its population rating. This reproducible rule identified 115 records; five of them proved eligible on full-text assessment and are included in the present version of the review, and the remaining 110 exclusions were confirmed. The record-level ratings, the re-examination set, and the revised decisions are given in Supplementary File S2. Of the 1000 records screened, 985 were excluded at the title and abstract stage and 15 reports were sought for retrieval; all 15 were obtained and assessed in full-text against the eligibility criteria. One of these reports was a systematic review and was therefore not counted as an included study, being retained as a background source only; 14 primary association studies were included in the synthesis. Criterion level ratings and the screening decision for every record are provided as Supplementary File S2, and the excluded records are summarized by the criterion that was not met in Supplementary Table S2. The selection process and the corresponding counts are shown in Figure 1.

### 2.5. Data Extraction

For each included study, the following were extracted into a standardized form: first author, year, country, design, sport and discipline, competitive level, sample sizes for athletes and comparison groups, sex, ancestry, gene and variant, genotyping method and Hardy–Weinberg status, personality instrument and dimensions, the reported genotype–phenotype association (test statistic, *p*-value, and η^2^/variance where given), direction of effect, and reported statistical power. The specific data items sought from each study are listed in Supplementary File S4 (Section S4.2). Data were extracted into the standardized form by two reviewers (K.Ł. and J.D.) working independently and in duplicate; discrepancies were resolved via consensus, with a third reviewer consulted where agreement could not be reached. Where genotype group means, standard deviations, or sample sizes required for effect-size computation were not given in the text, the published tables were reexamined, and quantities that remained unavailable were recorded as such; study authors were not contacted for additional data. All effect sizes reported in this review were computed exclusively from descriptive statistics printed in the source publications; no value was supplied by an author, imputed, or obtained from unpublished data. The extracted study-level data populate Table 2.

### 2.6. Handling of Overlapping Samples

Several Polish studies report recurring sample sizes (notably ~258 combat/martial arts athletes with ~278–284 controls) and overlapping authorship, indicating that the same participants may have been genotyped for different variants across publications. Records sharing a probable cohort were flagged (Table 2). Per protocol, studies sharing a cohort must be collapsed to a single independent observation per gene *×* trait contrast before any quantitative pooling, to avoid double counting participants.

### 2.7. Planned Effect-Size Harmonization

The pre-specified plan was to convert all personality outcomes to the standardized mean difference (Hedges’ *g*) under a harmonized allele carrier versus non-carrier genetic model (e.g., long allele/7R carriers vs. non-carriers; Met carriers vs. Val/Val; Del carriers vs. Ins/Ins), applying the small-sample correction. Computing g requires genotype group means, standard deviations, and sample sizes (or convertible statistics from two group contrasts).

#### Feasibility Outcome

On extraction, the eligible studies reported associations as multi-way ANOVA F statistics, η^2^, and allele frequencies. Critically, however, the full-text descriptive tables of eight of the fourteen primary studies do report genotype group means and standard deviations alongside the factorial ANOVA results. For these studies, carrier subgroup means and SDs were combined under the harmonized model and Hedges’ *g* with 95% confidence intervals was computed directly (Section 2.9; Figure 2). Two constraints nonetheless prevent conventional pooling. First, the reported genotype means are frequently collapsed across the athlete and control arms (the studies report a pooled genotype column rather than athlete only and control only genotype means), so several g values describe a whole sample genotype contrast rather than a within-athlete contrast; this is stated wherever it applies. Second, and decisively, the studies from the Polish research network share overlapping athletes and recurring control samples, so no gene *×* trait cell contains two genuinely independent estimates; the two studies conducted outside that network (in Egypt and Turkey) examine different instruments and do not provide a second estimate for any cell already covered. The number of cells supporting an independent sample random-effects meta-analysis is therefore zero, and no pooled estimate of any kind is reported (Section 2.9).

### 2.8. Quality and Risk-of-Bias Assessment

Methodological quality was appraised with a STREGA informed checklist covering sample size and power, recruitment and selection bias, blinding of genotyping and personality assessment, validation of sport participation and level, ancestry/population stratification control, Hardy–Weinberg reporting, multiple comparison correction, and completeness of effect size reporting. The appraisal was carried out by two reviewers (K.Ł. and J.D.) working independently, with disagreements resolved via consensus; no study was excluded on the basis of its rating, and the ratings inform the certainty assessment described in Section 2.11. The item-level appraisal is summarized in Table 5.

### 2.9. Synthesis Methods

Four complementary syntheses were undertaken. First, for every study reporting genotype group means and SDs, the standardized mean difference (Hedges’ *g*, with the small-sample correction) was computed for the allele-carrier-versus-non-carrier contrast in R (version 4.6.1; R Foundation for Statistical Computing, Vienna, Austria) with the base-R code provided as Script S1, and all values were cross-checked against the escalc function of the metafor package (version 5.0-1) [31], with carrier subgroups being combined using the standard formula for pooling subgroup means and variances; study-level g values with 95% confidence intervals are displayed as a forest plot (Figure 2), drawn with Matplotlib (version 3.10.8) in Python (version 3.12.3). No pooled estimate and no summary diamond are displayed for any cell, because no cell contains two independent samples; where two non-independent cohorts report the same contrast, their estimates are shown side by side and their directional consistency is described in the text without being combined. Second, a structured narrative synthesis is organized by gene and trait. Third was a vote counting synthesis of effect direction, reported following SWiM guidance: the number of associations reaching author-defined significance was tabulated, an exact binomial (sign) test was applied to the directional concordance of the most frequently studied variant, and the proportion of positive versus null primary reports was examined as an informal indicator of selective reporting (Figure 3). Fourth, for variants reporting athlete–control genotype distributions, an allele/genotype frequency analysis was performed by reconstructing genotype counts from reported percentages and computing odds ratios with 95% confidence intervals under allele, carrier, and recessive models (Figure 4); pooling across studies was undertaken only where ≥2 independent studies provided reconstructable counts. Heterogeneity statistics and funnel-based bias tests are not reported, because no cell accrues the independent sample count required for their valid computation.

### 2.10. Conditional Pooling (For Future Use, Once Independent Cohorts Exist)

Standardized mean differences have been computed for all cells where genotype-group means and SDs were recoverable (Section 2.9). The full conditional pooling protocol that would apply once independent cohorts become available, together with the per-study list of descriptive statistics still required, is provided as Supplementary File S4 in order to keep the Methods focused on the analyses actually performed. The analysis script accompanying the manuscript (Supplementary Script S1) recomputed every study-level Hedges’ *g* reported here from the genotype group means, standard deviations, and sample sizes printed in the source publications, collapses confirmed overlapping cohorts to single observations, and the minimum evidence rule was applied so that a summary estimate is generated only for cells with two or more independent samples; under the present evidence base, no cell meets that rule, and the script therefore returns study-level estimates only. The genotype group descriptive statistics underlying these computations are provided as Data S1.

### 2.11. Certainty Assessment

Certainty of the cumulative evidence was assessed for each gene *×* trait association using the Venice interim criteria developed by the Human Genome Epidemiology Network for genetic association studies, which grade three dimensions, the amount of evidence, consistency of replication, and protection from bias, as A, B, or C, and combine them into an overall rating of strong, moderate, or weak cumulative epidemiological credibility [32]. The amount of evidence was graded by the number of participants contributing to the least frequent genotype group; replication by the number of independent, non-overlapping samples reporting the same gene *×* trait contrast and the consistency of their direction; and protection from bias by the risk of bias appraisal (Section 2.8), the presence or absence of correction for multiple comparisons, the completeness of effect size reporting, and indications of selective reporting. Because all included studies were observational genetic association studies with modest samples, generally without correction for multiple comparisons, certainty was additionally expressed in GRADE terms, beginning at low certainty for observational evidence and rated down for risk of bias, imprecision, and suspected publication or selective reporting bias [33]. Certainty was assessed by two reviewers (K.Ł. and A.M.) independently, with disagreements resolved via discussion.

## 3. Results

### 3.1. Study Selection

The database searches returned 1245 records, of which 1000 entered title and abstract screening. Screening excluded 985 records, and 15 reports were sought for retrieval; all 15 were obtained and assessed in full-text (Figure 1). One report was excluded at the full-text stage as a secondary (review) article, in accordance with the prespecified eligibility criteria, and is cited as a background source only; fourteen primary genetic association studies were included in the review. Genotype group means and standard deviations permitting the computation of Hedges’ *g* were recoverable for eight of these fourteen studies (Section 3.5.1); no gene × trait cell, however, contained two independent, non-overlapping samples, so no pooled estimate is reported. All fourteen included studies contributed to the structured narrative synthesis, and the eleven of them that reported a testable genotype × trait contrast contributed to the vote-counting synthesis (Section 3.5.2). No report proved unobtainable.

### 3.2. Characteristics of Included Studies

The included studies are summarized in Table 2. The evidence base is predominantly male, Caucasian, and drawn from Polish combat-sport cohorts, with three studies conducted outside that research network: two in Turkey (one on wrestlers and badminton players, one on university students participating in risky and non-risky sports) and one on Egyptian athletes. An external systematic review of combat-sport genetics is summarized alongside the primary studies in Table 2 but was retained as a background source and contributed no primary data. Sample sizes for primary association studies ranged from 85 to 258 athletes. Personality was most often assessed with the Revised Temperament and Character Inventory (TCI-R; Cloninger’s model [7]) or the NEO Five Factor Inventory (NEO-FFI [1]); anxiety was assessed with the State Trait Anxiety Inventory (STAI [34]), impulsivity once with the Barratt Impulsiveness Scale 11 (BIS-11 [35]), and one study used the Freiburg Personality Inventory Revised (FPI-R [36]). *DRD2* rs1799732 was the most frequently examined single variant (three primary studies) and the *DAT1*/*SLC6A3* VNTR the most frequently examined repeat polymorphism (four primary studies), followed by single primary studies of *COMT* rs4680, the *DRD4* exon III VNTR, and further *DRD2* markers (rs6276, rs1800498, rs1079597), plus one multivariate cluster analysis.

### 3.3. Quality Assessment

Across studies, common strengths were Hardy–Weinberg testing and ancestry restriction to Caucasian participants to limit population stratification (Table 5). Recurrent limitations were the absence of blinding, incomplete or absent reporting of genotype group descriptive statistics, limited or absent multiple comparison correction, modest sample sizes, and a predominant reliance on male athletes from a single research network, raising concerns about both statistical power and the independence of cohorts across publications. Two studies deviated from these patterns in ways that matter for the synthesis: one applied a Bonferroni-corrected family-wise threshold and reported a Hardy–Weinberg deviation in its athlete group [25], and the two studies conducted outside the originating network reported neither the Hardy–Weinberg status nor ancestry controls [27,28].

### 3.4. Structured Synthesis by Gene and Trait

The reported associations are detailed below by gene and variant; their direction and statistical significance are summarized in Figure 3. All statistics are as reported by the original authors.

#### 3.4.1. *DRD2* rs1799732 (−141C Ins/Del)

Three primary studies examined this promoter variant. In sub-elite MMA athletes, a significant genotype effect on reward dependence (F(2,363) = 3.11, *p* = 0.0458, η^2^ = 0.017) and cooperativeness (F(2,363) = 3.24, *p* = 0.0402, η^2^ = 0.018) was reported, with a genotype × athletic status interaction for harm avoidance and cooperativeness and a direction of reduced harm avoidance and reward dependence in athletes. In a martial arts cohort, the −/− genotype was associated with lower reward dependence (F(1,536) = 3.95, *p* = 0.0472, η^2^ = 0.007), alongside effects on self-directedness, cooperativeness, and self-transcendence. In elite mixed martial arts athletes, ins/ins carriers showed the highest conscientiousness and del/del the lowest (F(1,319) = 7.38, *p* = 0.0069), with the genotype conscientiousness relationship moderated by athletic involvement. Three further *DRD2* markers were examined in the same research network. In the cohort of 141 athletes and 182 controls also reported by Lachowicz et al. [16], the exon 8 variant rs6276 showed a genotype × group interaction for extraversion (F(2,317) = 5.13, *p* = 0.0064, η^2^ = 0.031) and a nominal main effect of genotype on agreeableness (F(2,317) = 3.88, *p* = 0.0216, η^2^ = 0.024), the latter not surviving the authors’ Bonferroni-adjusted threshold; no genotype effect was found for neuroticism, openness, or conscientiousness, and the athlete group deviated from the Hardy–Weinberg equilibrium [25]. In 163 professional athletes and 232 controls, the Taq1B variant rs1079597 differed in genotype and allele frequency between athletes and controls; the result of the factorial analysis relating genotype to NEO-FFI dimensions could not be extracted from the record available to us [26]. A further study of the Taq1D variant rs1800498 in 159 athletes and 232 controls reported higher extraversion and conscientiousness in athletes [23]. The two TCI-R cohorts that report sample sizes consistent with a shared participant pool should be treated as non-independent (Table 2).

#### 3.4.2. *COMT* rs4680 (Val158Met)

One primary study of 258 combat-sport athletes and 278 controls reported significant associations of rs4680 with novelty seeking (F(2,530) = 5.958, *p* = 0.0028, η^2^ = 0.022) and self-management/self-directedness (F(2,530) = 6.772, *p* = 0.001), with the G/G genotype linked to lower novelty seeking and higher self-management in athletes relative to controls. This finding is directionally consistent with a role for the prefrontal dopamine tone in approach-related temperament, but rests on a single cohort.

#### 3.4.3. *DRD4* Exon-III VNTR

One primary study of 200 elite combat athletes and 102 controls reported significant group × *DRD4* genotype interactions for openness (*p* = 0.007; ~5.2% variance) and conscientiousness (*p* = 0.016; ~3.4% variance), with the s/s genotype linked to lower openness than the s/L genotype. Combat athletes scored higher on conscientiousness and lower on openness than controls. In the independent Turkish student sample described above, no significant *DRD4* effect was found on any Big Five dimension [28]. Notably, the historically prominent *DRD4* 7R–novelty seeking association was not the reported finding in either sample, consistent with the attenuated or null *DRD4* novelty seeking effects reported in general population meta-analyses [10,13,37] and with the failure of candidate-gene hypotheses to replicate in large genome-wide samples [38].

#### 3.4.4. *DAT1*/*SLC6A3* VNTR

One primary study of 100 elite combat athletes and 109 controls reported that elevated *DAT1* promoter methylation was associated with lower impulsivity (BIS-11), with a significant group × genotype interaction for methylation by athletic status; athletes showed lower impulsivity than controls [15]. The study reports genotype group means and standard deviations for the BIS-11 subscales but no test statistics for the genotype × trait contrast; the finding implicates an epigenetic rather than a purely sequence-level mechanism. Three further studies examined the VNTR itself. In 200 elite combat athletes and 102 controls, genotype frequencies differed between athletes and controls (χ^2^ = 6.79, *p* = 0.033) and genotype × group interactions were reported for state anxiety (F(2,296) = 3.55, *p* = 0.03), neuroticism (F(2,296) = 6.95, *p* = 0.001), agreeableness (F(2,296) = 6.44, *p* = 0.002), and conscientiousness (F(2,296) = 8.14, *p* = 0.0004); this sample is the same combat-athlete cohort reported for *DRD4* and in the cluster analysis [24]. Outside the Polish network, a study of 100 Egyptian athletes and 100 non-athletes reported higher 9/10 and 10/10 *SLC6A3* genotype frequencies in athletes but no significant genotype-related differences in Freiburg Personality Inventory Revised trait scores [27], and a study of 193 Turkish university students participating in risky and non-risky sports reported that non 10/10 carriers scored higher on agreeableness, with no significant *DAT1* effect on the remaining Big Five dimensions [28]. These are the only two included cohorts recruited outside the Polish research network for a candidate-variant contrast, and both are predominantly null.

#### 3.4.5. *ANKK1*/*DRD2* Marker Sets and Cluster Phenotypes

A cluster-based analysis of 200 elite combat athletes [21] mapped three psychological profiles (varying in anxiety, impulsivity, extraversion, and conscientiousness) onto dopamine-related variants including *DRD2* rs1076560, rs1800498, rs6276, *ANKK1* rs1800497, the *DAT1* VNTR, and the *DRD4* exon III VNTR, with significant genotype frequency differences across clusters (e.g., *DRD2* rs1076560 χ^2^ = 58.167, *p* < 0.0001, Bonferroni-corrected) but no non-athlete control group. A separate study of elite wrestlers and badminton players [22] linked *ANKK1*/*DRD2* markers (rs7118900, rs4436578) to neuroticism and exercise addiction, without reported effect sizes; a genome-wide association study of exercise addiction in elite Turkish wrestlers from the same research group identified only suggestively significant loci, none of them in dopaminergic genes [39], which illustrates the distance between candidate-gene and genome-wide evidence in this population. A further study of the *DRD2* Taq1D rs1800498 polymorphism [23] reported higher extraversion and conscientiousness in athletes than in controls; the sample is described as athletes without specification of the combat sport status (Table 2).

### 3.5. Quantitative Synthesis

Standardized mean differences (Hedges’ *g*) were computed for every study reporting genotype group means and SDs (eight of fourteen primary studies; Section 2.9). These study-level estimates are presented first; no pooled estimate is reported, because no gene × trait cell contains two independent samples. Two further syntheses that the data support are then reported: a vote counting synthesis of effect direction (following SWiM guidance) and an allele/genotype frequency analysis for the one variant with recoverable genotype counts. No funnel-based bias test or heterogeneity decomposition is reported, because no cell reaches the independent sample count these require.

#### 3.5.1. Standardized Mean Differences (Hedges’ *g*)

Genotype group means and standard deviations were recoverable from eight studies, permitting a direct computation of Hedges’ *g* for the allele-carrier-versus-non-carrier contrast; all 29 contrasts were computed from descriptive statistics printed in the source publications. The most frequently studied cell was *DRD2* rs1799732 with reward dependence, reported by two cohorts using the TCI-R. Del allele carriers scored lower on reward dependence in both (Niewczas et al. 2021 [17], g = −0.18, 95% CI −0.44 to +0.09; Chmielowiec et al. 2021 [20], g = −0.20, 95% CI −0.40 to +0.01), but each interval includes the null, and the two cohorts share the same control sample and overlapping athletes. The two estimates are therefore reported side by side as a single, directionally concordant but non-independent body of evidence and are not combined: pooling them would double count the same participants and produce a confidence interval that excludes the null on the strength of a single cohort. Their agreement in direction is described here as directional concordance, not as replication.

Across the remaining cells, single-study standardized effects were small. For *COMT* rs4680, Met allele carriers showed modestly higher novelty seeking (g = +0.29, 95% CI +0.01 to +0.58), an interval whose lower bound lies at the null, with negligible effects on harm avoidance, reward dependence, and self-directedness. For the *DRD4* exon III VNTR, long allele carriers scored higher on openness than s/s homozygotes (g = +0.22, 95% CI −0.02 to +0.45). For the *DAT1* VNTR, all BIS-11 contrasts between 9R carriers and 10R/10R homozygotes were near zero (|g| ≤ 0.19), whereas in the larger *DAT1* cohort, 9R carriers scored lower on neuroticism (g = −0.33, 95% CI −0.55 to −0.10), agreeableness (g = −0.26, 95% CI −0.49 to −0.04), and state anxiety (g = −0.29, 95% CI −0.51 to −0.06). For *DRD2* rs6276, G allele carriers scored slightly higher on agreeableness (g = +0.23, 95% CI +0.01 to +0.45), with all remaining NEO-FFI contrasts compatible with the null. A larger but cohort-specific effect was observed for *DRD2* rs1799732 with NEO-FFI conscientiousness, where Del allele carriers scored substantially lower than Ins/Ins homozygotes (g = −0.66, 95% CI −1.05 to −0.27); this estimate is derived from a different instrument, and a separate, though same-network, cohort and cannot be combined with the TCI-R reward dependence estimates. All study-level effects are displayed together in Figure 2. Of the 29 computable contrasts, 23 have confidence intervals that include the null, and no cell contains two independent samples, so no summary estimate is shown.

#### 3.5.2. Vote Counting Synthesis (Effect Direction)

Across the corpus, 21 of 26 reported gene × personality trait contrasts reached the significance threshold defined by the original authors. These contrasts were contributed by eleven of the fourteen included primary studies: *DRD2* rs1799732 with reward dependence and cooperativeness [17], with reward dependence, self-directedness, cooperativeness and self-transcendence [20], and with conscientiousness [16]; *COMT* rs4680 with novelty seeking and self-directedness [19]; the *DRD4* exon III VNTR with openness and conscientiousness [18]; the *DAT1* VNTR with impulsivity [15]; *ANKK1*/*DRD2* markers with neuroticism [22]; *DRD2* rs6276 with extraversion and agreeableness, with null contrasts for neuroticism, openness, and conscientiousness [25]; and the *DAT1* VNTR with neuroticism, agreeableness, conscientiousness, and state anxiety [24]. Two of the three studies conducted outside the Polish research network contributed predominantly null contrasts: no significant genotype-related differences in personality scores in Egyptian athletes [27], and a single significant *DAT1* agreeableness contrast against null results for *DRD4* and for the remaining Big Five dimensions in Turkish students [28]. Three included studies contributed no vote: the cluster phenotype analysis [21], which tested genotype frequencies across psychological clusters rather than a genotype × trait contrast and included no non-athlete comparison group; the *DRD2* Taq1D study [23], which reported athlete–control allele and genotype frequency differences without test statistics for a genotype × trait association; and the *DRD2* Taq1B study [26], for which the result of the genotype × trait analysis could not be extracted from the record available to us (Figure 3). For the most frequently studied variant, *DRD2* rs1799732, all three reporting studies showed a directionally concordant genotype effect (Del or −/− carriers exhibiting lower reward-dependence-related scores; exact sign test *p* = 0.25 for three concordant results), although at least two of these three studies probably share a cohort and cannot be treated as independent, so the sign test is reported for completeness only and does not test replication. The reporting pattern differs markedly by research network, and this contrast is informative. Every study from the Polish network that contributed a testable contrast reported at least one statistically significant association, whereas the two independent cohorts recruited outside it reported predominantly null genotype personality results. Given the modest samples (85–258 athletes) and the small effect sizes typical of candidate-gene personality associations, this asymmetry is more consistent with selective reporting within a single network than with a uniformly replicable effect (Section 4.3).

#### 3.5.3. Allele and Genotype Frequency Analysis (*COMT* rs4680)

Of the studies reporting athlete–control genotype distributions, only Humińska-Lisowska et al. [19] provided frequencies from which genotype counts could be reconstructed for a core variant with a validated personality outcome (258 combat sport athletes vs. 278 controls). Under an allele model, the *COMT* rs4680 A (Met) allele was significantly less frequent in athletes than controls (odds ratio 0.75, 95% CI 0.59–0.96), with a consistent recessive model estimate (A/A vs. G/G: OR 0.58, 95% CI 0.36–0.95) and a non-significant carrier model estimate (Met carrier vs. Val/Val: OR 0.85, 95% CI 0.57–1.27) (Figure 4). Other primary studies reported athlete–control genotype differences for markers outside the pre-specified core set (*DAT1* VNTR, χ^2^ = 6.79, *p* = 0.033 [24]; *SLC6A3* VNTR [27]; *DRD2* Taq1B rs1079597 [26]), while the three *DRD2* rs1799732 studies, the *DRD4* VNTR study, and the *DRD2* rs6276 study explicitly reported no significant athlete–control differences in genotype or allele frequency; none of these could be combined, because the contributing cohorts either overlap or examine different markers. As a single study result, the *COMT* estimate is presented for completeness and is not a meta-analytic pooled effect.

#### 3.5.4. Analyses That Could Not Be Performed

Pre-planned subgroup analyses (combat vs. non-combat vs. controls; elite vs. amateur; striking vs. grappling vs. mixed), independent sample random-effects pooling, heterogeneity quantification (I^2^, τ^2^), and small-study/publication bias tests (contour enhanced funnel plots, Egger’s regression) could not be validly executed, because they require a sufficient number of independent samples per cell, which the present evidence base does not provide: although standardized mean differences could be computed for most core variants (Section 3.5.1), no gene × trait cell contains two non-overlapping cohorts. Reported trait association effects, where quantified by the original authors, were small (η^2^ ≈ 0.003–0.048). Study-level standardized mean differences are presented in Figure 2; no pooled Hedges’ *g* and no funnel-based bias test is presented, because the cohort overlap and the absence of independent replication would make such estimates misleading. What is now required to complete a credible quantitative synthesis is not additional descriptive statistics but genuinely independent cohorts, as detailed in Supplementary File S4.

### 3.6. Certainty of the Cumulative Evidence

Cumulative epidemiological credibility was weak for every gene × trait association examined (Table 6). No association reached grade A or B for replication, because no cell contained two independent, non-overlapping samples reporting the same contrast: the two TCI-R cohorts reporting *DRD2* rs1799732 with reward dependence share the same control sample and overlapping athletes [17,20], and every remaining association rests on a single cohort. Protection from bias was graded C throughout, reflecting the absence of blinding, the general absence of correction for multiple comparisons, incomplete athlete-stratified descriptive reporting, and a reporting pattern in which every primary study from the originating research network with an extractable genotype × trait test reported at least one statistically significant association, while the independent cohorts did not (Section 3.5.2). The amount of evidence was graded C throughout: the Venice criteria grade this dimension by the number of participants in the least-frequent genotype group, and none of the included reports provides that count for the contrasts synthesized here, so the threshold of 100 participants required for grade B cannot be demonstrated for any association. In GRADE terms, certainty in the association between dopaminergic polymorphisms and sport-relevant personality traits is very low: the evidence begins at low certainty as observational data and is rated down further for risk of bias, imprecision (confidence intervals compatible with no effect in most cells), and suspected publication or selective reporting bias. The findings of this review are therefore hypothesis generating, and no clinical, selection, or talent identification inference should be drawn from them.

## 4. Discussion

### 4.1. Summary of Evidence

This review synthesized fourteen primary studies relating dopaminergic gene polymorphisms to validated personality measures in athletes, with a strong combat-sport emphasis. The most frequently reported signal was for *DRD2* rs1799732, where two cohorts reported lower reward dependence and reduced harm avoidance in −/− carriers [17,20] and a third reported higher conscientiousness in ins/ins carriers [16]; because these cohorts overlap, this consistency reflects repeated analysis of a shared participant pool rather than replication. Single cohort signals were reported for *COMT* rs4680 (novelty seeking, self-management, self-transcendence) [19], the *DRD4* exon-III VNTR (openness, conscientiousness) [18], further *DRD2* markers (rs6276, rs1079597, rs1800498) [23,25,26], and the *DAT1* VNTR, both as a sequence variant [24] and through promoter methylation [15]. Reported effects were small, consistent with the broader candidate-gene literature [10]. The two cohorts recruited outside the originating research network reported predominantly null associations [27,28], which is the single most important qualification based on the pattern above.

### 4.2. Interpretation in Light of the Dopaminergic Framework

Two further considerations qualify this interpretation. First, contemporary accounts of mesocorticolimbic dopamine emphasize its activation rather than its purely hedonic functions, namely behavioral activation, the exertion of effort, and effort-based cost–benefit decision making, which map less directly onto the reward dependence and novelty seeking constructs used throughout the present corpus than the original psychobiological model assumed [40]. Second, the variants examined here sit within a much broader sports genomics landscape in which the catalogue of markers associated with athlete status has expanded rapidly while individual effect sizes have remained uniformly small [41,42]. Central nervous system genes, including *DRD2* and *COMT*, have been proposed as plausible contributors to elite athletic traits only comparatively recently and largely on theoretical grounds [43,44]. Reviews restricted to combat-sport athletes reach a convergent conclusion: most reported associations concern physiological rather than psychological phenotypes, and the psychological subset rests on few, small, and rarely replicated samples [4,45].

### 4.3. Scope and Limits of the Quantitative Synthesis

Standardized mean differences could be computed for most core variants and are reported as study-level effects (Section 3.5.1), but a credible pooled meta-analysis of trait associations remains out of reach, for two principal reasons. The first is non-independence: recurring sample sizes, shared control groups, and overlapping authorship indicate that the studies from the originating research network draw on a shared cohort, so treating them as independent, for example by pooling the two TCI-R reward-dependence estimates [17,20], would double count the same participants and spuriously narrow confidence intervals [46]. This is why the present version reports no pooled estimate: in an earlier version of this manuscript a random effects estimate of that cell was displayed for transparency, and although it was labeled as anticonservative, a summary diamond whose interval excludes the null invites exactly the inference the data cannot support. The second is sparsity: even setting overlap aside, no gene × trait cell reaches two independent samples with compatible outcomes and instruments, so heterogeneity and small-study bias cannot be assessed [47,48]. A reporting issue compounds these: several studies report genotype means collapsed across athletes and controls rather than separately, so some computed effects describe a whole-sample genotype contrast rather than a within-athlete one; these estimates are identified as such in Figure 2 and in Data S1. A further interpretive caution arises from the vote counting result itself: that every study from the originating network with an extractable genotype × trait test reported at least one significant association while the two independent cohorts reported predominantly null results is difficult to reconcile with the small effect sizes and modest samples involved and is more plausibly a signature of selective reporting than of uniform replicability [10]. Presenting a pooled estimate, or funnel-based bias tests, under these conditions would imply a quantitative evidence base that does not yet exist. This position is consistent with the broader re-evaluation of candidate-gene research, in which historically prominent associations have generally failed to survive adequately powered replication and in which the apparent internal consistency of early literature has been attributed to limited power combined with selective reporting rather than to true effects [49]. Quantitative syntheses in sport genetics illustrate the same pattern: pooled estimates are typically small and heterogeneous even for the most intensively studied performance variants, and they attenuate as sample sizes increase [50,51].

### 4.4. Strengths and Limitations of This Review

This section brings together all limitations of the evidence base and of the review process; Section 4.3 above is confined to the limitations of the quantitative synthesis. Strengths include adherence to PRISMA 2020 and STREGA [30,52], transparent vote counting reported under SWiM guidance [53], explicit handling of probable cohort overlap, formal appraisal of the certainty of the cumulative evidence (Section 2.11 and Section 3.6), and provision of a ready-to-run quantitative synthesis protocol and script for when adequate data become available. Limitations of the evidence base reflect the underlying literature. It is narrow, predominantly male, largely single-country and, decisively, largely produced by one research network, so that repeated reports do not constitute independent replication and no gene × trait association reaches two non-overlapping samples. The predominance of male participants is not peculiar to this literature. Audits of sport and exercise science journals report that approximately one third of participants are female and that only about six per cent of studies are conducted exclusively in women [54,55], and molecular work in exercise science has shown that the skeletal muscle response to an identical training stimulus, at both the protein and the DNA methylation level, differs by sex [56]. Conclusions drawn from the present corpus should therefore be regarded as provisional for female athletes.

Instruments and genetic models are heterogeneous (TCI-R, NEO-FFI, STAI, BIS-11, and FPI-R operationalize overlapping but non-identical constructs; studies apply dominant, recessive, additive, and carrier contrasts), several studies examine different markers within the same gene, and effect size reporting is incomplete, with genotype means frequently collapsed across athletes and controls. Eligibility was restricted by design to five dopaminergic genes, so variants in other dopaminergic genes (for example *DRD1*, *DRD3*, *DRD5*, *DBH*, *TH*, and *MAOA*) and non-dopaminergic candidates fall outside the scope of this synthesis. Limitations of the review process itself are that title and abstract screening was automation-assisted and initially applied the population criterion more narrowly than the protocol specified, an error identified and corrected during peer review via full-text re-examination of all records rated positively on the variant and outcome domains (Section 2.4); that no unpublished data were sought from authors; that several authors of this review are authors of included studies, mitigated by the safeguards described in the Conflicts of Interest statement; and that the certainty of the cumulative evidence was weak for every association examined (Section 3.6).

While the current observations are biologically plausible, they must be contextualized within modern behavioral genetics. Personality traits are now recognized as highly polygenic phenotypes, with individual common variants accounting for only a small fraction of phenotypic variance. Indeed, large-scale genome-wide association studies (GWASs) demonstrate that behavioral phenotypes emerge from the collective, cumulative impact of thousands of genetic loci rather than isolated candidate genes. For this reason, identified associations involving *DRD2*, *DRD4*, *COMT*, or *DAT1* should be viewed as preliminary and hypothesis-generating rather than conclusive evidence of causal genetic mechanisms. To comprehensively elucidate the genetic architecture of athlete personality, subsequent research should adopt integrative frameworks that combine genome-wide methodologies, polygenic scores, and environmental variables [38,57,58]. Contemporary genome-wide analyses reinforce this point. The most recent large-scale meta-analysis of the five factor traits identified 208 independent loci for neuroticism but only 14, 7, 3, and 2 for extraversion, openness, agreeableness, and conscientiousness, respectively, with individual variant effects that are uniformly minute [59]. Even where polygenic indices are constructed from data of this scale, their predictive accuracy varies appreciably with the characteristics of the samples in which they are derived and applied, which counsels caution before importing them into athlete populations [60].

Therefore, the present review should be interpreted as summarizing evidence from the candidate-gene era, while highlighting the need for independent replication using contemporary genomic approaches.

### 4.5. Beyond Static Candidate-Gene Associations: Epigenetic and Polygenic Perspectives

A limitation of the present evidence base that is conceptual rather than statistical is that every included study treats genotype as a fixed antecedent of a fixed trait. Dopaminergic signaling is, however, regulated dynamically, and the included corpus itself contains the clearest illustration of this. The only study to examine an epigenetic mechanism reported that elevated methylation of the *DAT1* promoter, rather than the VNTR genotype, tracked lower impulsivity in elite combat athletes, with a genotype × athletic status interaction for methylation [15]. The sequence variant alone was uninformative in the same sample, and our own standardized estimates for the BIS-11 contrasts were near zero (Figure 2). Two further reports from the same network describe higher *DAT1* promoter methylation in athletes than in controls. Taken together, these observations suggest that the regulatory state of a dopaminergic gene may carry information that its sequence does not.

This matters for interpretation because prolonged training is itself an environmental exposure capable of inducing epigenetic remodeling, an inference now well supported in skeletal muscle, where acute exercise and chronic training alter DNA methylation at promoters, gene bodies, enhancers, and intergenic regions [61], where individuals with more than fifteen years of endurance or resistance training differ from untrained controls in the baseline methylation of exercise-responsive genes [62], and where these responses are themselves sex-dependent [56]. If methylation differences between athletes and controls are partly training-induced, then cross-sectional athlete–control contrasts cannot separate a predisposition that led an individual into combat sport from an adaptation acquired through years of practice. The included studies are all cross-sectional, and none measured training exposure longitudinally, so the classical question of whether athletes are born or made cannot be addressed with this evidence, in either direction. We therefore avoid any developmental or causal reading of the associations summarized above.

The same caution applies on the genomic scale. Personality is highly polygenic, and large genome-wide studies attribute only a small fraction of phenotypic variance to any individual common variant [57,58]; candidate-gene hypotheses of the kind examined here have repeatedly failed to replicate when tested in adequately powered samples [38]. Consistent with this, the one genome-wide association study conducted in an athletic population within our search window identified only suggestively significant loci, none of them in dopaminergic genes [39]. Future work in this area will need to combine polygenic scores with epigenetic profiling, the longitudinal measurement of training exposure, and gene–environment interaction models. Until such designs exist, the associations reviewed here should be treated as candidate-era hypotheses about dopaminergic contributions to sport-relevant temperament, not as established genotype–phenotype relationships.

The wider behavioral genetic literature offers a comparable caution. Where dopaminergic candidate effects on risk-related phenotypes have been examined in populations recruited outside Europe and outside sport, the *DRD4* exon-III 7R allele has been associated with risk tolerance but not with novelty seeking [63], and *DRD2* Taq1A has been associated with extraversion rather than with the reward-dependence constructs emphasized here [64]. Independent athlete cohorts examining adjacent monoaminergic systems likewise report psychometric associations that do not converge on a single dopaminergic account [65]. Taken together with the null results of the two non-network cohorts included in this review [27,28], these findings suggest that the coherence of the Polish literature is at least partly an artefact of its shared provenance.

### 4.6. Implications and Future Directions

Primary studies should report genotype group descriptive statistics (means, SDs, n) for each personality outcome and adopt a harmonized carrier-versus-non-carrier model to enable future synthesis [29,52]. Independent, multicenter cohorts with diverse sex representation are needed to establish whether the *DRD2* rs1799732 signal generalizes beyond the existing Polish samples [4,39]. Larger designs incorporating polygenic scores, gene–environment interaction, epigenetic profiling, and longitudinal follow-up would help disentangle selection (athletes self-selecting into combat sport) from training-induced adaptation and from genuine genotype–trait associations [2].

Such designs would also allow sport-relevant temperament to be modeled alongside the psychological variables that are already known to track competitive performance, for which meta-analytic estimates exist and against which any genetic increment should be benchmarked [66,67]. Finally, because associations of this kind are routinely translated into commercial talent identification products well ahead of the evidence, any future claim of predictive validity should be evaluated against the explicit standards that have been proposed for genetic testing in sport [68,69].

## 5. Conclusions

Across fourteen eligible primary studies, dopaminergic gene polymorphisms, most frequently *DRD2* rs1799732 and the *DAT1*/*SLC6A3* VNTR and, in single cohorts, *COMT* rs4680 and the *DRD4* exon-III VNTR, were reported as associated with sport-relevant personality traits in athletes, predominantly in combat sport. Where the source tables permitted, these associations were quantified as standardized mean differences (Hedges’ *g*), which were uniformly small and, in the great majority of contrasts, compatible with no effect; for the most studied *DRD2* rs1799732 × reward-dependence cell, the two reporting cohorts were directionally concordant but individually non-significant, and they were not pooled. The decisive limitation is no longer missing descriptive statistics but the non-independence of the evidence: the contributing cohorts overlap and share controls, so no gene × trait association is yet supported by replication in two independent samples, and the two cohorts recruited outside the originating research network reported predominantly null associations. The principal contribution of this review is therefore to quantify the existing associations transparently, to show why a pooled estimate is not yet justified, and to specify the design improvements, above all independent, multicenter cohorts with diverse sex representation, alongside athlete-stratified genotype group statistics, harmonized genetic models, and the integration of polygenic and epigenetic measures, which would make a credible meta-analysis possible in the future.

## Figures and Tables

**Figure 1:**
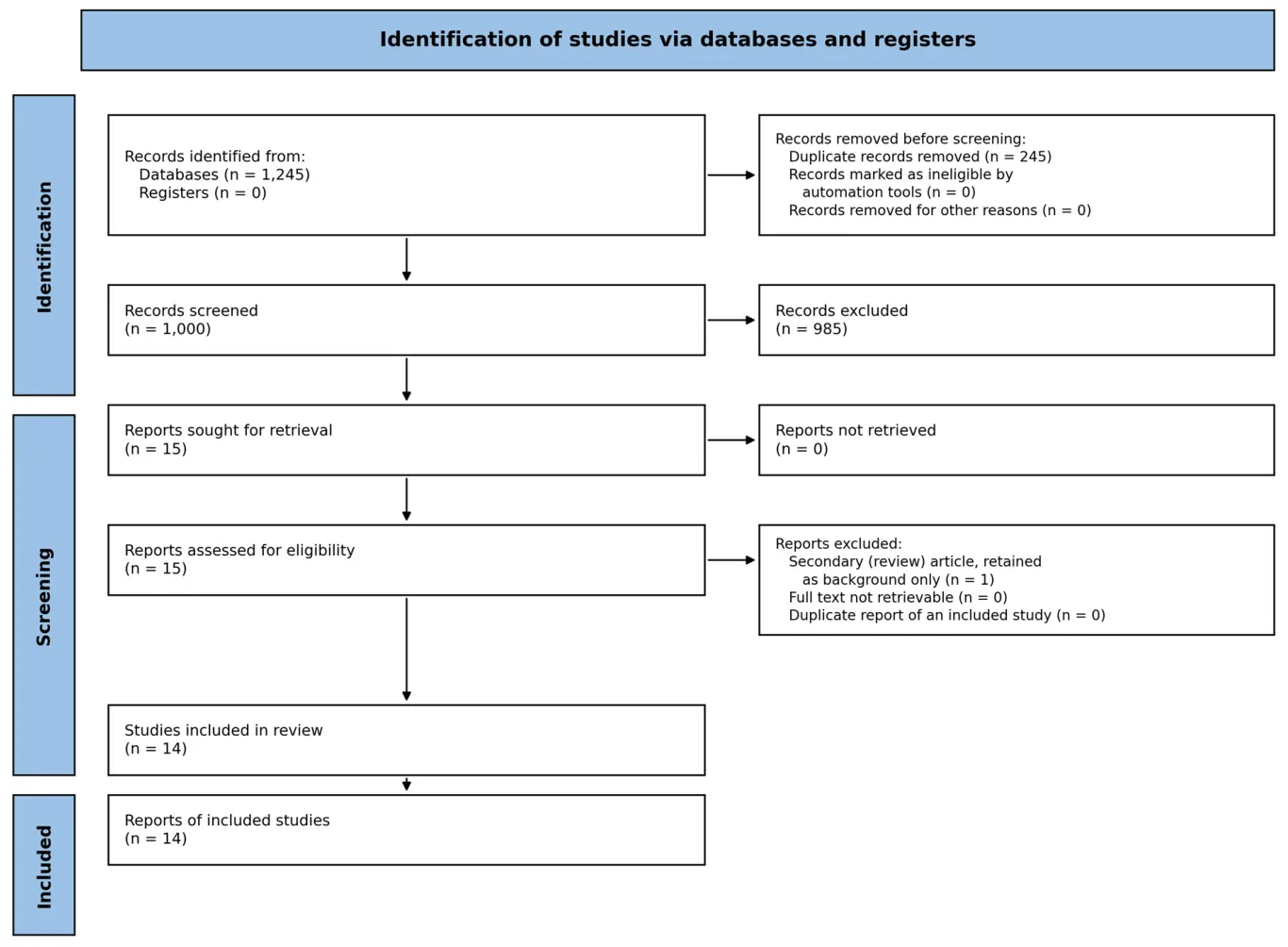
PRISMA 2020 flow diagram of the identification, screening, and selection of studies [30]. Records were identified using five electronic databases (PubMed, Scopus, Web of Science Core Collection, SPORTDiscus, and Embase). Of the 1245 records identified, 245 duplicates were removed, 1000 records were screened, 15 reports were assessed in full text, and 14 primary studies were included. Reasons for exclusion are summarized in Supplementary Table S2. Five of the 15 reports assessed in full-text were identified through the extended verification described in Section 2.4.

**Figure 2:**
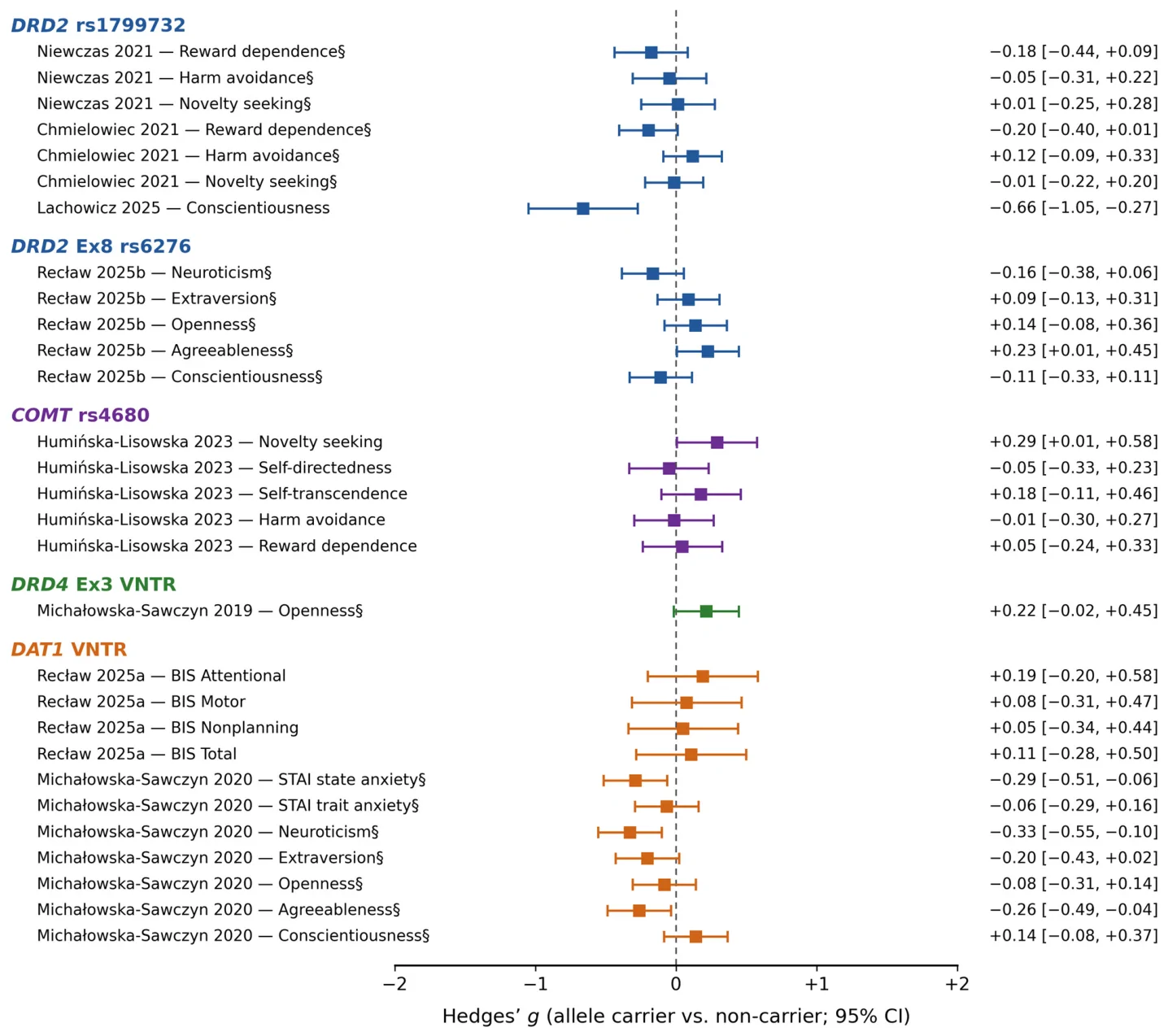
Forest plot of study-level standardized mean differences (Hedges’ *g*) for allele carriers versus non-carriers across the included studies, grouped by gene and variant. Squares indicate point estimates, and horizontal lines indicate 95% confidence intervals; the dashed line indicates the null. Each estimate was calculated from genotype-group means, standard deviations, and sample sizes reported in the source publication [15,16,17,18,19,20,24,25]. § indicates contrasts computed for the combined athlete and control sample rather than for athletes alone. No pooled estimates are shown because no gene × trait cell contained two independent samples.

**Figure 3:**
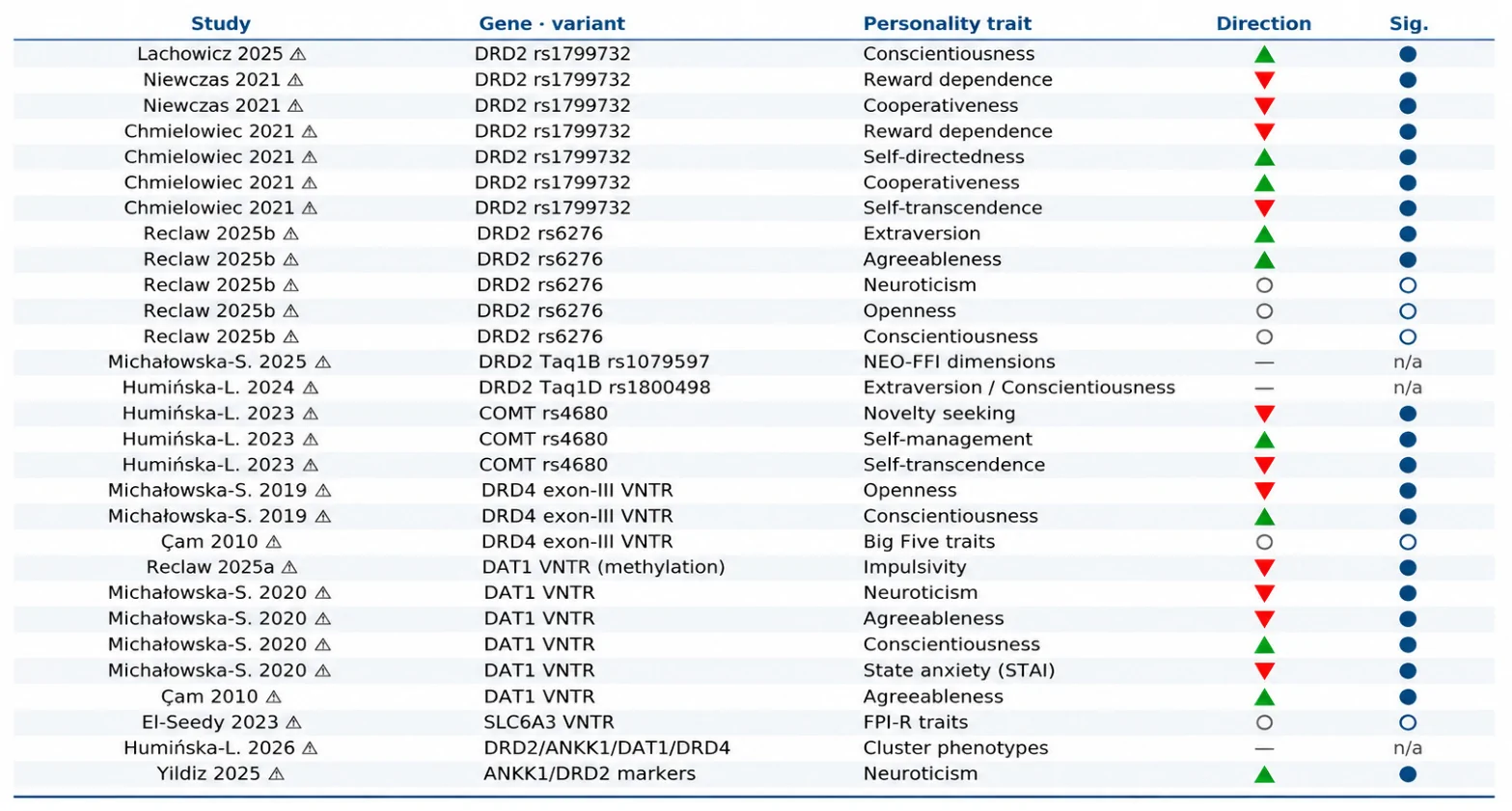
Effect direction plot of reported genotype–personality contrasts across the fourteen included primary studies. ▲ indicates a higher trait score and ▼ a lower trait score in the carrier or minor allele group relative to the reference genotype, green and red symbols indicate higher and lower trait scores, respectively; ○ indicates no significant genotype effect reported by the original authors; ● indicates a statistically significant contrast reported by the original authors; n/a indicates that no testable genotype–trait contrast was reported or could be extracted from the available record; ⚠ indicates a probable shared cohort based on recurring sample sizes and overlapping authorship [15,16,17,18,19,20,21,22,23,24,25,26,27,28].

**Figure 4:**
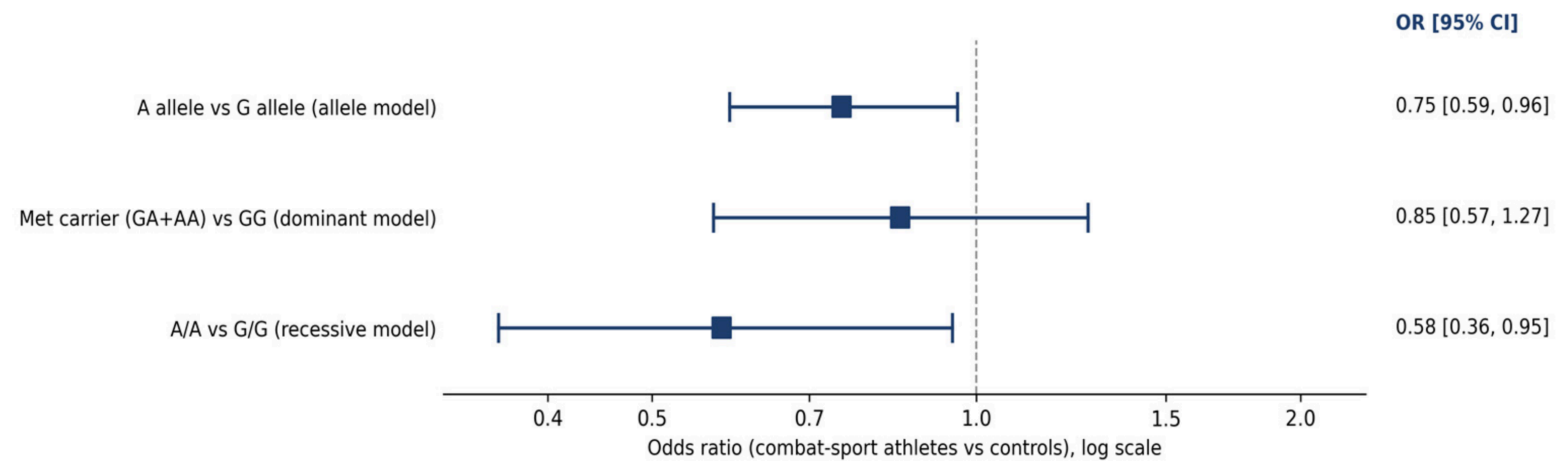
*COMT* rs4680 odds ratios for combat-sport athletes versus controls under three genetic models, from a single study (Humińska-Lisowska et al., 2023 [19]; 258 athletes vs. 278 controls; genotype counts reconstructed from reported frequencies). Squares are point estimates and horizontal lines are 95% confidence intervals; the dashed line marks the null (OR = 1). This is a single study estimate, not a pooled meta-analysis.

**Table 1:** The five primary objectives of the review, the evidence available for each, and the section addressing it. The more specific questions registered in the protocol are treated as secondary analyses and are reported within the sections listed.

Research Question	Evidence Status in This Review	Addressed in
O1. Which dopaminergic variants have been studied in relation to personality traits in athletes?	Across 14 primary studies; reported effects were small and largely null in the two independent cohorts	Section 3.4, Section 3.5.1 and Section 4.1
O2. Which personality traits are most frequently implicated, and what are the reported effect sizes?	Reward dependence, harm avoidance, conscientiousness, novelty seeking, agreeableness, neuroticism; 29 standardized effects computed	Section 3.4 and Section 3.5.1
O3. Do associations differ by competitive level, sport category, or personality instrument?	Not analyzable (insufficient independent data)	Section 3.5
O4. Is there evidence of selective reporting or small-study bias?	Indirect: all studies from the originating network positive, both independent cohorts largely null; funnel tests not feasible	Section 3.5.1 and Section 4.3
O5. Is the evidence sufficiently independent and consistently reported to support quantitative pooling, and what is needed for a credible meta-analysis?	Study-level g computed for eight studies (29 contrasts); no pooling possible (no cell has two independent samples); vote counting and a single study OR were performed	Section 2.7, Section 2.9, Section 3.5, Section 4.3 and Section 5

**Table 2:** Characteristics and main reported findings of the fourteen included primary studies and one systematic review retained as a background source. Statistics are reported as presented in the original publications.

Study (Author, Year)	Venue/DOI	Gene · Variant	Sport · Level	Athletes/Controls	Instrument	Trait(s)	Reported Association (as Published)
Lachowicz M et al. 2025 [16]	Genes · 10.3390/genes16060720	*DRD2* · rs1799732 (−141C Ins/Del)	Karate, judo, boxing, MMA, jiu-jitsu, kickboxing · Elite/professional	141/182 (92 M, 49 F)	NEO-FFI	Conscientiousness	ins/ins genotype carriers showed the highest Conscientiousness and del/del the lowest; significant genotype effect (F(1,319) = 7.38, *p* = 0.0069), moderated by athletic group membership.
Niewczas M et al. 2021 [17] ⚠ possible shared cohort	Genes · 10.3390/genes12081217	*DRD2* · rs1799732 (−141C Ins/Del)	MMA · Sub-elite (≥8 yr training; international competitors)	85/284 (Male)	TCI-R	Reward dependence, harm avoidance, cooperativeness	Significant genotype effect on reward dependence (F(2,363) = 3.11, *p* = 0.0458, η^2^ = 0.017) and cooperativeness (F(2,363) = 3.24, *p* = 0.0402, η^2^ = 0.018); genotype × athletic status interaction for harm avoidance and cooperativeness. Direction: reduced harm avoidance and reward dependence in athletes.
Michałowska-Sawczyn M et al. 2019 [18]	Biology of Sport · 10.5114/biolsport.2019.85457	*DRD4* · Exon-III VNTR (s/L)	Combat athletes (discipline not specified) · Elite	200/102 (Male)	NEO-FFI, STAI	Openness, conscientiousness	Significant group × *DRD4* genotype interactions for openness (*p* = 0.007; 5.2% variance) and conscientiousness (*p* = 0.016; 3.4% variance). s/s genotype linked to lower openness vs. s/L. Combat athletes scored higher on conscientiousness (7.18 vs. 5.98) and lower on openness (4.42 vs. 4.63) than controls.
Humińska-Lisowska K et al. 2023 [19] ⚠ possible shared cohort	Journal of Human Kinetics · 10.5114/jhk/168789	*COMT* · rs4680 (Val158Met)	MMA (85), judo (53), boxing (51), karate (21), kickboxing (34), wrestling (14) · Sub-elite (≥5 yr training)	258/278 (Male)	TCI-R	Novelty seeking, self-directedness, self-transcendence	Significant genotype effects on novelty seeking (F(2,530) = 5.958, *p* = 0.0028, η^2^ = 0.022) and self-management/self-directedness (F(2,530) = 6.772, *p* = 0.001). G/G genotype linked to lower novelty seeking and higher self-management in athletes vs. controls.
Chmielowiec K et al. 2021 [20] ⚠ possible shared cohort	Genes · 10.3390/genes12081219	*DRD2* · rs1799732 (−141C Ins/Del)	Martial arts (discipline not specified) · Not specified	258/284 (Male)	TCI-R	Reward dependence, self-directedness, cooperativeness, self-transcendence	*DRD2* rs1799732 −/− associated with lower reward dependence (F(1,536) = 3.95, *p* = 0.0472, η^2^ = 0.007), and effects on self-directedness (F(1,536) = 4.42, *p* = 0.0358, η^2^ = 0.008), cooperativeness (F(2,536) = 3.94, *p* = 0.0201, η^2^ = 0.014) and self-transcendence (F(2,536) = 3.79, *p* = 0.0231, η^2^ = 0.013).
Humińska-Lisowska K et al. 2026 [21]	Biology · 10.3390/biology15030290	*DRD2*, *ANKK1*, *DAT1*, *DRD4* · *DRD2* rs1076560, rs1800498, rs6276, rs1799732; *ANKK1* rs1800497; *DAT1* VNTR; *DRD4* exon-III VNTR	Judo (51), wrestling (38), boxing (50), kickboxing (32), karate (29) · Top-elite (16), elite (68), sub-elite (116)	200/0 (Male)	NEO-FFI	Anxiety, impulsivity, extraversion, conscientiousness (cluster phenotypes)	Three psychological clusters mapped onto dopamine-related variants (e.g., *DRD2* rs1076560 χ^2^ = 58.167, *p* < 0.0001; Bonferroni corrected). Cluster 1: higher anxiety/impulsivity; Cluster 2: higher extraversion/conscientiousness with lower anxiety/impulsivity. No non-athlete control group.
Yıldız YA et al. 2025 [22]	International Journal of Sport and Exercise Psychology · 10.1080/1612197x.2025.2584537	*ANKK1*, *DRD2* · rs7118900, rs4436578, rs2283265, rs1125394	Wrestling (combat); badminton (comparator) · Elite	68/39 (Not reported)	Exercise addiction/personality (not fully specified in record)	Neuroticism (and exercise addiction)	rs7118900 and rs4436578 associated with exercise addiction; rs7118900 and rs4436578 linked to neuroticism. Effect sizes and *p*-values not reported in the available record.
Humińska-Lisowska K et al. 2024 [23]	Baltic Journal of Health and Physical Activity · 10.29359/BJHPA.16.1.09	*DRD2* · Taq1D rs1800498	Athletes (combat sport status not specified in record) · Not specified	159/232 (Male)	NEO-FFI	Extraversion, Conscientiousness	*DRD2* Taq1D rs1800498 associated with higher extraversion and conscientiousness in athletes vs. controls; significant allele/genotype frequency differences reported. Effect sizes and *p*-values not reported in the available record.
Anastasiou K et al. 2024 [4]	International Journal of Environmental Research and Public Health · 10.3390/ijerph21081019	*DRD2*, *ANKK1* (review) · *DRD2* rs1079597, rs1799732; *ANKK1* rs1800497	Striking (956), grappling (1274), MMA (437) · High-level (national/international/Olympic)	3323/0 (Mixed)	Review (no primary personality instrument)	Personality/psychological determinants (review-level)	Systematic review identifying *DRD2* rs1079597 and rs1799732 among variants associated with psychological determinants in combat athletes; minor allele direction noted as positive for combat-sport success. No primary effect sizes; included here as a background systematic review, not as a primary data source for synthesis.
Recław R et al. 2025a [15]	Biomedicines · 10.3390/biomedicines13122893	*DAT1*/*SLC6A3* · 40-bp VNTR (+ promoter methylation)	Combat athletes (discipline not specified) · Elite	100/109 (Male)	BIS-11	Impulsivity	Elevated *DAT1* promoter methylation associated with lower impulsivity (BIS-11) in elite athletes; significant group × genotype interaction for methylation by athletic status. Athletes showed lower impulsivity than controls. Effect sizes and *p*-values not reported in the available record.
Michałowska-Sawczyn M et al. 2020 [24] ⚠ possible shared cohort	Journal of Human Kinetics · 10.2478/hukin-2019-0119	*DAT1*/*SLC6A3* · 40-bp VNTR (9R/10R)	Judo (51), wrestling (38), boxing (50), kickboxing (32), karate (29) · Top -elite (16), elite (68), sub-elite (69)	200/102 (Male)	NEO-FFI, STAI	Neuroticism, agreeableness, conscientiousness, state anxiety	Genotype frequencies differed between athletes and controls (χ^2^ = 6.79, *p* = 0.033). Group × genotype interactions for state anxiety (F(2,296) = 3.55, *p* = 0.03), neuroticism (F(2,296) = 6.95, *p* = 0.001), agreeableness (F(2,296) = 6.44, *p* = 0.002), and conscientiousness (F(2,296) = 8.14, *p* = 0.0004). Same combat-athlete cohort as Michałowska-Sawczyn 2019 [18] and Humińska-Lisowska 2026 [21].
Recław R et al. 2025b [25] ⚠ possible shared cohort	Brain Sciences · 10.3390/brainsci15090965	*DRD2* · rs6276 (Ex8)	Karate, judo, boxing, MMA, ju-jitsu, kickboxing, plus team sports · National/international	141/182 (92 M, 49 F)	NEO-FFI	Extraversion, agreeableness, conscientiousness	Genotype × group interaction for extraversion (F(2,317) = 5.13, *p* = 0.0064, η^2^ = 0.031); nominal main effect of genotype on agreeableness (F(2,317) = 3.88, *p* = 0.0216), not surviving the authors’ Bonferroni threshold; no genotype effect for neuroticism, openness, or conscientiousness. Athlete group deviated from Hardy–Weinberg equilibrium. Same cohort as Lachowicz 2025 [16].
Michałowska-Sawczyn M et al. 2025 [26] ⚠ possible shared cohort	Biology of Sport · 10.5114/biolsport.2025.139470	*DRD2* · Taq1B rs1079597	Professional athletes (disciplines not specified) · Professional	163/232 (114 M, 49 F athletes)	NEO-FFI, MINI	NEO-FFI personality dimensions	Statistically significant differences in *DRD2* Taq1B rs1079597 genotype and allele frequencies between professional athletes and controls (chi-square). Genotype × NEO-FFI results and genotype-group means and SDs not extractable from the retrieved record.
El-Seedy A et al. 2023 [27]	Archives of Molecular Biology and Genetics · 10.33696/genetics.2.012	*DAT1*/*SLC6A3* · 3′-UTR 40-bp VNTR	Individual and team sports (Egypt) · Not specified	100/100 (50 M, 50 F)	FPI-R	Neuroticism, aggressiveness, and further FPI-R scales	9/10 and 10/10 genotypes significantly more frequent in athletes than controls; no statistically significant genotype-related differences in personality trait scores were reported. Independent cohort, recruited outside the Polish research network.
Çam F et al. 2010 [28]	Türkiye Klinikleri Journal of Medical Sciences · 10.5336/medsci.2009-14839	*DRD4* · exon-III VNTR; *DAT1*/*SLC6A3* · 40-bp VNTR (5-HT2A also examined, outside scope)	Risky and non-risky sports (college students, Turkey) · Recreational/collegiate	193 (risky and non-risky sport participants; no non-athlete control)	FFPI	Big Five dimensions	Non 10/10 *DAT1* carriers scored higher on agreeableness than 10/10 homozygotes; no significant *DAT1* effect on the remaining Big Five dimensions and no significant *DRD4* effect on any dimension. Independent cohort, recruited outside the Polish research network.

⚠ flags studies whose recurring sample sizes and overlapping authorship indicate a probable shared cohort; such studies must be collapsed to a single independent observation per gene × trait contrast before any future quantitative pooling. “Anastasiou et al. 2024” [4] is an external systematic review included as background, not as a primary data source.

**Table 3:** PICOS eligibility framework.

Element	Criterion
Population	Combat sport or broader athletic populations; human; validated personality assessment
Exposure	≥1 core dopaminergic polymorphism (*DRD2*, *DRD4*, *COMT*, *DAT1*/*SLC6A3*, *ANKK1*)
Comparison	Genotype groups (carrier vs. non-carrier/between genotypes) and/or athlete vs. control
Outcomes	Personality/temperament dimensions (e.g., novelty seeking, reward dependence, harm avoidance, conscientiousness, impulsivity, openness, extraversion, anxiety)
Study design	Case control, cohort or cross-sectional with a comparison structure; reviews retained as background only

PICOS framework used to define inclusion and exclusion.

**Table 4:** Boolean search strategy (applied across PubMed, Scopus, Web of Science, SportDiscus, and Embase).

Concept Block	Search Terms (OR Within Block; AND Between Blocks)
Dopaminergic genes	“dopamine” OR “dopaminergic” OR *DRD2* OR *DRD4* OR *COMT* OR *DAT1* OR *SLC6A3* OR *ANKK1*
Personality	“personality” OR “novelty seeking” OR “sensation seeking” OR “impulsivity” OR “reward dependence” OR “harm avoidance” OR “conscientiousness” OR “temperament” OR “Big Five” OR TCI OR NEO
Population	“combat sport*” OR “martial art*” OR “fight sport*” OR “contact sport*” OR “athlete*” OR MMA OR boxing OR judo OR wrestling OR karate OR taekwondo OR kickboxing
Limits	Human; peer reviewed; original data; validated personality instrument

The asterisk (*) denotes a truncation/wildcard operator used to retrieve different word endings sharing the same word stem. Full, database-specific strings as executed, together with the date of each search and the number of records retrieved from each source, are provided in Supplementary Table S1.

**Table 5:** STREGA-informed quality and risk of bias summary of the fourteen included primary studies.

Study	HWE Reported	Ancestry Control	Power Reported	Multiple Test Correction	Genotype-Group M ± SD Reported (Athlete Stratified?)	Blinding
Lachowicz M 2025 [16]	Yes	Caucasian only	No	Not reported	Yes	Not reported
Niewczas M 2021 [17]	Yes	Caucasian only	Yes	Not reported	Yes	Not reported
Michałowska-Sawczyn M 2019 [18]	Yes	Caucasian only	No	Not reported	Yes	Not reported
Humińska-Lisowska K 2023 [19]	Yes	Caucasian only	No	Not reported	Yes	Not reported
Chmielowiec K 2021 [20]	Yes	Caucasian only	Yes	Not reported	Yes	Not reported
Humińska-Lisowska K 2026 [21]	No	Caucasian only	No	Yes (Bonferroni)	No	Not reported
Yıldız YA 2025 [22]	No	Caucasian only	No	Not reported	No	Not reported
Humińska-Lisowska K 2024 [23]	No	Caucasian only	No	Not reported	No	Not reported
Recław R 2025 [15]	No	Caucasian only	No	Not reported	Yes	Not reported
Michałowska-Sawczyn M 2020 [24]	Yes	Caucasian only	No	Not reported	Yes (whole sample columns; not athlete stratified)	Not reported
Recław R 2025b (rs6276) [25]	Yes (controls); athletes deviate	Caucasian only	Yes	Yes (Bonferroni)	Yes (genotype × group cells)	Not reported
Michałowska-Sawczyn M 2025 [26]	Yes	Caucasian only	No	Not reported	No	Not reported
El-Seedy A 2023 [27]	Not reported	Egyptian sample; no stratification control	No	Not reported	No	Not reported
Çam F 2010 [28]	Not reported	Turkish sample; no stratification control	No	Not reported	No	Not reported

Genotype group means and standard deviations were recoverable for eight of the fourteen primary studies and were used to compute study-level Hedges’ *g* (Section 3.5.1); the remaining six did not report them. The final column distinguishes two situations that were previously conflated: studies that report genotype group means and SDs only for the whole sample, pooled across athletes and controls, and studies that report them separately by athletic status. Only the latter permit a strictly within-athlete contrast; contrasts derived from whole sample columns are flagged in Figure 2 and in Data S1. Incomplete athlete-stratified descriptive reporting and, decisively, the non-independence of the contributing cohorts are the principal reasons an independent sample pooled meta-analysis was not feasible.

**Table 6:** Certainty of the cumulative evidence for each gene *×* trait association (Venice criteria: A, B, or C for amount of evidence, replication, and protection from bias; overall rating strong, moderate, or weak).

Gene × Trait Association	Independent Samples (k)	Amount of Evidence (Least Frequent Genotype Group)	Replication	Protection from Bias	Venice Grade	GRADE Certainty
*DRD2* rs1799732 × reward dependence (TCI-R)	0 (two overlapping cohorts)	C	C	C	Weak	Very low
*DRD2* rs1799732 × conscientiousness (NEO-FFI)	1	C	C	C	Weak	Very low
*COMT* rs4680 × novelty seeking/self-directedness	1	C	C	C	Weak	Very low
*DRD4* exon III VNTR × openness/conscientiousness	1	C	C	C	Weak	Very low
*DAT1* VNTR and methylation × impulsivity (BIS-11)	1	C	C	C	Weak	Very low
*ANKK1*/*DRD2* marker sets × cluster phenotypes	1	C	C	C	Weak	Very low
*COMT* rs4680 allele frequency, athletes vs. controls	1	C	C	C	Weak	Very low
*DRD2* rs6276 × extraversion/agreeableness (NEO-FFI)	1	C	C	C	Weak	Very low
*DRD2* Taq1B rs1079597 × NEO-FFI traits	1	C	C	C	Weak	Very low
*DAT1*/*SLC6A3* VNTR × neuroticism/agreeableness/conscientiousness/anxiety	2 (1 Polish, 1 Turkish; different instruments)	C	C	C	Weak	Very low
*SLC6A3* VNTR × FPI-R traits (independent cohort)	1	C	C	C	Weak	Very low

The amount of evidence is graded by the number of participants in the least-frequent genotype group (Venice: A, more than 1000; B, 100–1000; C, fewer than 100). None of the included reports gives that count for the contrasts synthesized here, so grade B cannot be demonstrated for any association and the dimension is graded C throughout; total sample sizes of the contributing cohorts ranged from 107 to 542 participants. Replication is graded C wherever no two independent, non-overlapping samples report the same contrast. Grading follows the Venice interim criteria [32]; GRADE certainty follows [33].

## Data Availability

The original contributions presented in this study are included in the article/Supplementary Materials. Further inquiries can be directed to the corresponding author.

## Supplementary Materials

The following supporting information can be downloaded at: https://www.mdpi.com/article/10.3390/ijms27198484/s1.

## Abbreviations

The following abbreviations are used in this manuscript:

*ANKK1*	Ankyrin Repeat and Kinase Domain Containing 1
BIS-11	Barratt Impulsiveness Scale 11
CI	Confidence Interval
*COMT*	Catechol O Methyltransferase
*DAT1*	Dopamine Transporter 1
*DRD2*	Dopamine Receptor D2
*DRD4*	Dopamine Receptor D4
HWE	Hardy-Weinberg Equilibrium
MMA	Mixed Martial Arts
NEO-FFI	NEO Five-Factor Inventory
OR	Odds Ratio
PICOS	Population, Intervention/Exposure, Comparison, Outcomes, Study Design
PRISMA	Preferred Reporting Items for Systematic Reviews and Meta-Analyses
SD	Standard Deviation
*SLC6A3*	Solute Carrier Family 6 Member 3
STAI	State Trait Anxiety Inventory
STREGA	Strengthening the Reporting of Genetic Association Studies
SWiM	Synthesis Without Meta-analysis
TCI	Temperament and Character Inventory
TCI-R	Temperament and Character Inventory Revised
VNTR	Variable Number Tandem Repeat
