# Supplementary material for: Dopaminergic Gene Polymorphisms and Personality Traits in Athletes: A Systematic Review and Exploratory Quantitative Synthesis
Source: Int J Mol Sci. 2026 Sep 23;27(19):8484. doi: 10.3390/ijms27198484 (PMC13665109; doi:10.3390/ijms27198484)
Supplement: Supplementary file 1 [file ijms-27-08484-s001.zip › File S1 PRISMA 2020 checklist.pdf]

File S1. PRISMA 2020 checklist (revised submission)

Dopaminergic Gene Polymorphisms and Personality Traits in Athletes: A Systematic Review and Exploratory Quantitative Synthesis

Item wording is abbreviated; the full wording is given in Page MJ, McKenzie JE, Bossuyt PM, et al. The PRISMA 2020 statement: an updated guideline for reporting systematic reviews. BMJ 2021;372:n71 (CC BY 4.0). Section numbers refer to the revised manuscript; page numbers are omitted because they will change at typesetting.

Section / topic	#	Checklist item (abbreviated)	Location where item is reported
TITLE			
Title	1	Identify the report as a systematic review	Title page (p. 1)
ABSTRACT			
Abstract	2	Structured abstract following PRISMA 2020 for Abstracts	Abstract (p. 1)
INTRODUCTION			
Rationale	3	Describe the rationale in the context of existing knowledge	§1.1–1.5
Objectives	4	State the objectives or questions the review addresses	Abstract (Objective); §1.6; Table 1 (five primary objectives O1–O5 with nested secondary questions)
METHODS			
Eligibility criteria	5	Inclusion and exclusion criteria and how studies were grouped	§2.2 and Table 3; rationale for the candidate-gene set in §1.4
Information sources	6	All databases and other sources searched, with the date each was last searched	§2.3: five databases, all last searched 15 March 2026; no registers, websites or citation searching; Table S1
Search strategy	7	Full search strategies, including filters and limits	§2.3 and Table 4; Table S1
Selection process	8	Number of reviewers, independence, and any automation tools used	§2.4: LLM-assisted title/abstract screening; two reviewers (K.Ł., J.D.) verified retained records and a 10% sample of exclusions; third reviewer (A.M.). During revision a systematic error in the automated population rating was identified and corrected by full-text re-examination of a reproducible set of 115 excluded records by two reviewers; five additional eligible studies were identified (§2.4; File S2)
Data collection process	9	Number of reviewers, independence, and any automation tools used	§2.5: two reviewers (K.Ł., J.D.) independently and in duplicate; consensus, third reviewer where needed; authors not contacted; all effect sizes computed from published tables only
Data items	10a	Outcomes sought and how results were selected within studies	§2.2, §2.5
	10b	All other variables sought and assumptions about missing data	§2.5
Study risk of bias	11	Tool used, number of assessors, and their independence	§2.8: STREGA-informed checklist, two independent assessors (K.Ł., J.D.); Table 5
Effect measures	12	Effect measure(s) used in the synthesis and presentation of results	§2.7, §2.9
Synthesis methods	13a	How studies were assigned to each synthesis	§2.6, §2.9
	13b	Data preparation and handling of missing summary statistics	§2.7, §2.9; Data S1; Script S1
	13c	Methods used to tabulate or visually display results	§2.9
	13d	Methods used to synthesise results and rationale; model and software	§2.9 (no pooling performed; rationale stated); conditional pooling protocol in File S4; effect sizes computed in base R (Script S1) and cross-checked with metafor

Section / topic	#	Checklist item (abbreviated)	Location where item is reported
	13e	Methods to explore heterogeneity	File S4; not performed, §3.5.4
	13f	Sensitivity analyses	File S4; not performed, §3.5.4
Reporting bias assessment	14	Methods used to assess risk of bias due to missing results	§2.9
Certainty assessment	15	Methods used to assess certainty in the body of evidence	§2.11: Venice interim criteria plus GRADE; two independent assessors (K.Ł., A.M.)
RESULTS			
Study selection	16a	Numbers screened, assessed and included, ideally with a flow diagram	§3.1; Figure 1 (1,245 identified; 1,000 screened; 985 excluded; 15 assessed; 14 included)
	16b	Studies that met many but not all criteria, with reasons for exclusion	§3.1; Figure 1; Table S2; File S2 (re-examination set)
Study characteristics	17	Cite each included study and present its characteristics	§3.2; Table 2 (fourteen primary studies, with reference numbers)
Risk of bias in studies	18	Risk-of-bias assessments for each included study	§3.3; Table 5
Results of individual studies	19	Summary statistics and effect estimates for each study	§3.4, §3.5.1; Figure 2 (29 study-level estimates); Figure 3; Data S1
Results of syntheses	20a	Characteristics and risk of bias of studies contributing to each synthesis	§3.3, §3.5.1
	20b	Results of each synthesis, with precision and direction of effect	§3.5.1–3.5.3; Figures 2–4 (no pooled estimate reported)
	20c	Results of investigations of heterogeneity	§3.5.4 — not estimable; reason given
	20d	Results of sensitivity analyses	§3.5.4 — not performed; reason given
Reporting biases	21	Risk of bias due to missing results in each synthesis	§3.5.2; §4.3
Certainty of evidence	22	Certainty in the body of evidence for each outcome assessed	§3.6 and Table 6
DISCUSSION			
Discussion	23a	General interpretation of the results in the context of other evidence	§4.1, §4.2, §4.5
	23b	Limitations of the evidence included in the review	§4.3 (quantitative synthesis); §4.4 (consolidated limitations)
	23c	Limitations of the review processes used	§4.4 (including the screening error identified and corrected during revision)
	23d	Implications for practice, policy and future research	§4.6; §6
OTHER INFORMATION			
Registration and protocol	24a	Registration name and number	§2.1 (PROSPERO CRD420261430605); Data Availability
	24b	Where the protocol can be accessed	§2.1
	24c	Amendments to the registered information or protocol	§2.1 (two deviations reported)
Support	25	Sources of financial and non-financial support	Funding
Competing interests	26	Competing interests of the review authors	Conflicts of Interest
Availability of data, code and other materials	27	Which materials are publicly available and where	Data Availability; File S1 (this checklist), File S2 (record-level screening decisions and re-examination set), File S3 (SWiM checklist), File S4 (conditional pooling protocol), Tables S1–S2, Data S1 with Script S1

Note on item 8. Title and abstract screening was supported by an automated, large language model-based tool. Peer review identified that the tool had applied the population criterion more narrowly than the registered eligibility criteria; the corrected procedure, the reproducible rule defining the 115 re-examined records, and the five studies added as a result are documented in §2.4 and in File S2.
