# Supplementary material for: Dopaminergic Gene Polymorphisms and Personality Traits in Athletes: A Systematic Review and Exploratory Quantitative Synthesis
Source: Int J Mol Sci. 2026 Sep 23;27(19):8484. doi: 10.3390/ijms27198484 (PMC13665109; doi:10.3390/ijms27198484)
Supplement: Supplementary file 1 [file ijms-27-08484-s001.zip › File S3 SWiM checklist.pdf]

File S3. SWiM (Synthesis Without Meta-analysis) reporting checklist (revised submission)

Dopaminergic Gene Polymorphisms and Personality Traits in Athletes: A Systematic Review and Exploratory Quantitative Synthesis

SWiM comprises nine reporting items and complements PRISMA. Items follow Campbell M, McKenzie JE, Sowden A, et al. BMJ 2020;368:l6890. Item wording is abbreviated; locations refer to sections of the revised manuscript.

SWiM item	Item description (abbreviated)	How it is addressed	Location
METHODS			
1a Grouping studies for synthesis	Description of, and rationale for, the groups used in the synthesis	Studies were grouped into gene × personality-trait cells (DRD2 rs1799732 and further DRD2 markers, COMT rs4680, DRD4 exon-III VNTR, DAT1/SLC6A3 VNTR, ANKK1/DRD2 marker sets), because the review question concerns genotype–phenotype association. Cells were sub-grouped by instrument (TCI-R, NEO-FFI, STAI, BIS-11, FPI-R), judged non-interchangeable, so only within-cell, within-instrument contrasts are treated as comparable	§2.6, §2.9, §3.4
1b Changes to the groups after the protocol	Detail and justify changes made subsequent to the protocol	The protocol planned random-effects pooling per gene × trait cell; because no cell contained two independent samples, cells were retained as reporting units but not pooled. Reported as a protocol deviation. Eligibility at the gene level admitted additional DRD2 markers (rs6276, rs1800498, rs1079597) during revision	§2.1, §2.7, §1.4
2 Standardised metric and transformation methods	Standardised metric for each outcome, why it was chosen, and any transformations	Hedges' g with the small-sample correction under a harmonised allele carrier versus non-carrier model; carrier subgroups combined with the standard formula for pooling subgroup means and variances; genotype × group cells pooled within genotype where a source reports athletes and controls separately. Odds ratios for allele and genotype frequency contrasts. All values recomputed from published tables by Script S1	§2.7, §2.9; Data S1; Script S1
3 Synthesis methods	Describe and justify the methods used when meta-analysis was not possible	Three complementary syntheses: (i) study-level standardised mean differences with 95% CIs, reported individually and never pooled (Figure 2); (ii) vote counting of effect direction with an exact binomial (sign) test for the most studied variant, presented with direction and significance in separate columns (Figure 3); (iii) allele/genotype frequency odds ratios for the one core variant with reconstructable counts (Figure 4). Pooling was withheld because the contributing cohorts are not independent	§2.9; §3.5.1–3.5.3
4 Criteria used to prioritise results	Criteria, with justification, used to select studies for the main synthesis or conclusions	A minimum-evidence rule: a summary estimate is displayed only for a cell with at least two independent, non-overlapping samples; no cell met that threshold and no pooled estimate is reported. Studies sharing a cohort are collapsed to one observation per cell. Funnel-based tests require at least ten independent samples per cell and were not attempted. A transparency-labelled pooled estimate shown in the first submitted version has been withdrawn	§2.6, §2.9; §3.5.1; §4.3; File S4
5 Investigation of heterogeneity in reported effects	Methods used to examine heterogeneity when meta-analysis was not possible	Heterogeneity was examined descriptively by comparing the direction and magnitude of study-level effects within each cell, and by tabulating instruments, genetic models, sample scope (whole-sample versus athlete-only contrasts) and cohort overlap. I^2 and τ^2 were pre-specified but are not reported, because their valid computation requires two independent samples per cell	§2.9; §3.5.4; File S4
6 Certainty of evidence	Methods used to assess certainty of the synthesis findings	Venice interim criteria for genetic associations (amount of evidence, replication, protection from bias), graded A/B/C and combined into strong, moderate or weak credibility, with GRADE in parallel; amount of evidence graded by the least frequent genotype group	§2.11; §3.6; Table 6

SWiM item	Item description (abbreviated)	How it is addressed	Location
RESULTS			
7 Data presentation methods	Graphical and tabular methods used to present the effects	Forest plot of all study-level estimates with whole-sample contrasts flagged (Figure 2), effect-direction plot with direction and significance in separate columns and the reference genotype named (Figure 3), odds-ratio plot (Figure 4), and Tables 2, 5 and 6 with reference numbers for every study. Every caption states what the display does and does not represent; no figure displays a pooled estimate	§2.9; Figures 2–4; Tables 2, 5, 6
8 Reporting results	Description of the synthesised findings and their certainty, indicating which studies contribute	Findings are reported per gene × trait cell, naming the eleven studies that contribute the 26 vote-counted contrasts (21 significant) and the three that contribute no vote, flagging shared cohorts, and distinguishing the originating research network from the two independent cohorts, whose contrasts were predominantly null. The certainty of each association is given in Table 6. Results are framed as hypothesis-generating; the text states that no clinical, selection or talent-identification inference should be drawn	§3.4, §3.5.1–3.5.3, §3.6
DISCUSSION			
9 Limitations of the synthesis	Limitations of the synthesis methods or groupings and how they affect the conclusions	Vote counting ignores magnitude and is sensitive to selective reporting; study-level standardised effects are not comparable across instruments and genetic models; several genotype means are collapsed across athletes and controls; cohort overlap precludes independent replication; all included studies are cross-sectional and cannot separate predisposition from training-induced adaptation. Conclusions are therefore hypothesis-generating	§4.3, §4.4, §4.5
