# Supplementary material for: Dopaminergic Gene Polymorphisms and Personality Traits in Athletes: A Systematic Review and Exploratory Quantitative Synthesis
Source: Int J Mol Sci. 2026 Sep 23;27(19):8484. doi: 10.3390/ijms27198484 (PMC13665109; doi:10.3390/ijms27198484)
Supplement: Supplementary file 1 [file ijms-27-08484-s001.zip › File S4 conditional pooling protocol.pdf]

File S4. Conditional pooling protocol and data required to complete a standardized-mean-difference meta-analysis

Dopaminergic Gene Polymorphisms and Personality Traits in Athletes: A Systematic Review and Exploratory Quantitative Synthesis

This file collects material that was part of the main text in the version first submitted (Section 2.10 and Appendix A) and was moved to the Supplementary Materials during revision, so that the Methods describe only the analyses actually performed. Nothing in this file was applied to the data in the present review: under the current evidence base no gene × trait cell contains two independent, non-overlapping samples, and no pooled estimate is reported. The protocol below specifies what would be done, and what would be needed, should independent cohorts become available.

S4.1 Conditional pooling protocol

Standardized mean differences (Hedges' g, allele carrier versus non-carrier, with the small-sample correction) have been computed for every gene × trait cell in which genotype-group means, standard deviations and sample sizes were recoverable from the published tables (main text, Section 2.9; Data S1). Should independent samples become available — that is, cohorts recruited outside the originating Polish research network that do not share its recurring control groups — the following would be performed per gene × trait cell containing at least two such independent samples:

(a) Random-effects meta-analysis using the restricted maximum likelihood (REML) estimator, with the DerSimonian–Laird estimator reported as a sensitivity analysis, implemented in the metafor package in R [22].

(b) Quantification of heterogeneity by I^2 and τ^2, with 95% confidence intervals for both.

(c) Prespecified subgroup analyses by sport category (combat versus non-combat), competitive level (elite versus sub-elite/amateur) and, within combat sport, striking versus grappling versus mixed disciplines, provided each subgroup contains at least two independent samples.

(d) Assessment of small-study effects by contour-enhanced funnel plots and Egger's regression test, applied only where a cell contains at least ten independent samples [25].

(e) Application of the minimum-evidence rule implemented in Script S1: cohorts confirmed to overlap are collapsed to a single observation per cell before pooling, and a summary estimate is generated only for cells that retain two or more independent samples after collapsing. Instruments are not interchanged: TCI-R, NEO-FFI, STAI, BIS-11 and FPI-R contrasts are pooled only within instrument.

(f) Effects are expressed under the harmonized carrier-versus-non-carrier model wherever the source permits (long-allele/7R carriers versus non-carriers for the DRD4 exon-III VNTR; Met carriers versus Val/Val for COMT rs4680; Del carriers versus Ins/Ins for DRD2 rs1799732; 9R carriers versus 10R/10R for the DAT1 VNTR; minor-allele carriers versus major-allele homozygotes for other DRD2 markers). Where a source reports genotype means only for the whole sample, pooled across athletes and controls, the resulting estimate describes a whole-sample contrast and is flagged as such (Data S1, column sample_scope).

S4.2 Per-study data required

Two further steps are required before a credible independent-sample pooled meta-analysis becomes defensible. First, for the existing studies, genotype-group means, standard deviations and n are needed separately for athletes and for controls; several studies report only a genotype column collapsed across both arms, so the corresponding contrasts in Data S1 are whole-sample rather than within-athlete. Second, and indispensably, genuinely independent cohorts must be added, because the present studies cannot be pooled without double-counting the same participants. The table below lists, for each included study, the specific data to extract or to request from the corresponding authors.

Study (reference; locator)	Specific data to extract or request	Current status in Data S1
Niewczas et al. 2021 [17] (Genes 12(8):1217)	Table 3 — TCI-R dimension means ± SD by DRD2 rs1799732 genotype (ins/ins, ins/del, del/del), for MMA athletes and controls separately; genotype counts per group.	Whole-sample contrasts computed (3)
Chmielowiec et al. 2021 [18] (Genes 12(8):1219)	Table 4 — TCI-R means ± SD by DRD2 rs1799732 genotype separately for athletes and controls; genotype counts. Confirm overlap with Niewczas 2021 and Humińska-Lisowska 2023 (shared controls).	Whole-sample contrasts computed (3)

Study (reference; locator)	Specific data to extract or request	Current status in Data S1
Lachowicz et al. 2025 [21] (Genes 16(6):720)	NEO-FFI means ± SD by DRD2 rs1799732 genotype for athletes and controls separately; genotype counts.	Athlete contrast computed (1)
Humińska-Lisowska et al. 2023 [19] (J Hum Kinet 89:89–99)	Table 4 already reports TCI-R means ± SD by COMT rs4680 genotype separately for athletes and controls; genotype counts from Table 2. Nothing further required.	Athlete contrasts computed (5)
Michałowska-Sawczyn et al. 2019 [20] (Biol Sport 36(4):365–372)	Table 4 — NEO-FFI/STAI sten means ± SD by DRD4 exon-III VNTR genotype (s/s, s/l, l/l), athletes and controls separately; genotype counts.	Whole-sample contrast computed (1)
Recław et al. 2025a [15] (Biomedicines 13(12):2893)	BIS-11 means ± SD by DAT1 VNTR genotype and by methylation stratum for athletes and controls; genotype counts.	Athlete contrasts computed (4)
Humińska-Lisowska et al. 2026 [34] (Biology 15(3):290)	NEO-FFI means ± SD by genotype for each reported variant. No non-athlete control group; usable for within-athlete genotype contrasts only.	Not computable (no genotype-group M ± SD)
Yıldız et al. 2025 [36] (Int J Sport Exerc Psychol, advance online)	Personality/neuroticism means ± SD by ANKK1/DRD2 genotype for wrestlers and badminton players; the specific instrument and its subscale statistics.	Not computable
Humińska-Lisowska et al. 2024 [35] (Balt J Health Phys Act 16(1):Article 9)	NEO-FFI means ± SD by DRD2 Taq1D rs1800498 genotype, athletes and controls separately; genotype counts; combat-sport status of the sample.	Not computable
Michałowska-Sawczyn et al. 2020 [44] (J Hum Kinet 72:79–89)	Table 3 reports STAI/NEO-FFI sten means ± SD by DAT1 VNTR genotype for the whole sample; athlete-only and control-only genotype columns are needed for a within-athlete contrast. Same cohort as [20] and [34].	Whole-sample contrasts computed (7)
Recław et al. 2025b [42] (Brain Sci 15(9):965)	Table 4 already reports NEO-FFI means ± SD by DRD2 rs6276 genotype separately for athletes and controls, with cell n in Table 2. Nothing further required. Same cohort as [21].	Contrasts computed from genotype × group cells (5)
Michałowska-Sawczyn et al. 2025 [43] (Biol Sport, doi 10.5114/biolsport.2025.139470)	NEO-FFI means ± SD by DRD2 Taq1B rs1079597 genotype, athletes and controls separately; the result of the factorial ANOVA relating genotype to NEO-FFI dimensions; genotype counts.	Not computable from the retrieved record
El-Seedy et al. 2023 [45] (Arch Mol Biol Genet 2(1):12–20)	FPI-R scale means ± SD by SLC6A3 VNTR genotype (9/9, 9/10, 10/10) for athletes; genotype counts for athletes and non-athletes.	Not computable from the retrieved record
Çam et al. 2010 [46] (Türkiye Klinikleri J Med Sci 30(5):1454–1460)	FFPI Big Five means ± SD by DAT1 VNTR (10/10 vs non-10/10) and by DRD4 exon-III VNTR genotype; genotype counts; separation of risky and non-risky sport participants.	Not computable from the retrieved record

Once athlete-stratified means, SDs and counts are confirmed, and once genuinely independent cohorts are added, Script S1 will recompute g per gene × trait contrast, collapse any confirmed overlapping cohorts to single independent observations, and pool only those cells with two or more independent samples. The external systematic review [4] is background only and is not a primary data source.
