# Supplementary material for: Dopaminergic Gene Polymorphisms and Personality Traits in Athletes: A Systematic Review and Exploratory Quantitative Synthesis
Source: Int J Mol Sci. 2026 Sep 23;27(19):8484. doi: 10.3390/ijms27198484 (PMC13665109; doi:10.3390/ijms27198484)
Supplement: Supplementary file 1 [file ijms-27-08484-s001.zip › Supplementary Tables S1 and S2.pdf]

Supplementary Materials — Tables S1 and S2 (revised submission)

Dopaminergic Gene Polymorphisms and Personality Traits in Athletes: A Systematic Review and Exploratory Quantitative Synthesis

Table S1. Search strategies as executed

All five databases were searched from inception to 15 March 2026. Limits applied in every database: human studies, peer-reviewed original reports, and studies using a validated personality or temperament instrument. No language or publication-year restriction was applied. The search itself was not repeated during revision; the changes in the revised submission concern screening decisions only (Table S2, File S2).

Database	Interface	Date searched	Records retrieved	Search field
PubMed	NLM	15 March 2026	312	[tiab]
Scopus	Elsevier	15 March 2026	398	TITLE-ABS-KEY
Web of Science Core Collection	Clarivate	15 March 2026	287	TS=
SPORTDiscus	EBSCOhost	15 March 2026	156	Keyword
Embase	Elsevier	15 March 2026	92	:ti,ab,kw
Total records identified			1,245	
Duplicate records removed before screening			245	
Records screened at title and abstract			1,000	

PubMed

((dopamine[tiab] OR dopaminergic[tiab] OR DRD2[tiab] OR DRD4[tiab] OR COMT[tiab] OR DAT1[tiab] OR SLC6A3[tiab] OR ANKK1[tiab])) AND ((personality[tiab] OR “novelty seeking”[tiab] OR “sensation seeking”[tiab] OR impulsivity[tiab] OR “reward dependence”[tiab] OR “harm avoidance”[tiab] OR conscientiousness[tiab] OR temperament[tiab] OR “Big Five”[tiab] OR TCI[tiab] OR NEO[tiab])) AND ((“combat sport*”[tiab] OR “martial art*”[tiab] OR “fight sport*”[tiab] OR “contact sport*”[tiab] OR athlete*[tiab] OR MMA[tiab] OR boxing[tiab] OR judo[tiab] OR wrestling[tiab] OR karate[tiab] OR taekwondo[tiab] OR kickboxing[tiab]))

Scopus

TITLE-ABS-KEY (dopamine OR dopaminergic OR DRD2 OR DRD4 OR COMT OR DAT1 OR SLC6A3 OR ANKK1) AND TITLE-ABS-KEY (personality OR “novelty seeking” OR “sensation seeking” OR impulsivity OR “reward dependence” OR “harm avoidance” OR conscientiousness OR temperament OR “Big Five” OR TCI OR NEO) AND TITLE-ABS-KEY (“combat sport*” OR “martial art*” OR “fight sport*” OR “contact sport*” OR athlete* OR MMA OR boxing OR judo OR wrestling OR karate OR taekwondo OR kickboxing)

Web of Science Core Collection

TS=(dopamine OR dopaminergic OR DRD2 OR DRD4 OR COMT OR DAT1 OR SLC6A3 OR ANKK1) AND TS=(personality OR “novelty seeking” OR “sensation seeking” OR impulsivity OR “reward dependence” OR “harm avoidance” OR conscientiousness OR temperament OR “Big Five” OR TCI OR NEO) AND TS=(“combat sport*” OR “martial art*” OR “fight sport*” OR “contact sport*” OR athlete* OR MMA OR boxing OR judo OR wrestling OR karate OR taekwondo OR kickboxing)

SPORTDiscus

(dopamine OR dopaminergic OR DRD2 OR DRD4 OR COMT OR DAT1 OR SLC6A3 OR ANKK1) AND (personality OR “novelty seeking” OR “sensation seeking” OR impulsivity OR “reward dependence” OR “harm avoidance” OR conscientiousness OR temperament OR “Big Five” OR TCI OR NEO) AND (“combat sport*” OR “martial art*” OR “fight sport*” OR “contact sport*” OR athlete* OR MMA OR boxing OR judo OR wrestling OR karate OR taekwondo OR kickboxing)

Embase

(dopamine:ti,ab,kw OR dopaminergic:ti,ab,kw OR DRD2:ti,ab,kw OR DRD4:ti,ab,kw OR COMT:ti,ab,kw OR DAT1:ti,ab,kw OR SLC6A3:ti,ab,kw OR ANKK1:ti,ab,kw) AND (personality:ti,ab,kw OR “novelty seeking”:ti,ab,kw OR “sensation seeking”:ti,ab,kw OR impulsivity:ti,ab,kw OR “reward dependence”:ti,ab,kw OR “harm avoidance”:ti,ab,kw OR conscientiousness:ti,ab,kw OR temperament:ti,ab,kw OR “Big Five”:ti,ab,kw OR TCI:ti,ab,kw OR NEO:ti,ab,kw) AND (“combat sport*”:ti,ab,kw OR “martial art*”:ti,ab,kw OR “fight sport*”:ti,ab,kw OR “contact sport*”:ti,ab,kw OR athlete*:ti,ab,kw OR MMA:ti,ab,kw OR boxing:ti,ab,kw OR judo:ti,ab,kw OR wrestling:ti,ab,kw OR karate:ti,ab,kw OR taekwondo:ti,ab,kw OR kickboxing:ti,ab,kw)

Table S2. Reasons for exclusion at title and abstract screening (n = 985)

Counts were computed directly from the record-level screening file (File S2, File_S2_screening_decisions.xlsx), in which every screened record carries a yes / maybe / no rating on each of the six eligibility domains together with its screening decision. In the revised submission the five records retrieved and included after the extended full-text

verification described in Section 2.4 are no longer counted as exclusions (n = 985, previously 990); four of them had carried no domain rated "no" and one had been rated "no" on the population domain. The figures below are reproducible from File S2 by tabulating the records whose Revised_decision does not begin with "Retrieved".

Panel A. Primary reason for exclusion (mutually exclusive)

Each excluded record is assigned to the first domain it failed, in the order shown. Percentages are of the excluded records and sum to 100%.

Domain not met (rated "no")	n	% of 985	Cumulative %
Not a human study	21	2.1	2.1
No athletic or combat-sport population	801	81.3	83.5
No dopaminergic polymorphism analyzed	35	3.6	87.0
No validated personality instrument	15	1.5	88.5
No genotype–personality association reported	0	0.0	88.5
Ineligible study design (review, editorial, comment)	1	0.1	88.6
No single domain rated "no"; excluded on the overall screening judgment	112	11.4	100.0
Total	985	100.0	

Panel B. Domains not met, in any combination (not mutually exclusive)

A record may fail several domains; the counts below therefore exceed the total and the percentages do not sum to 100%.

Domain rated "no"	n	% of 985	Rated "no" or "maybe" (n)
Athletic or combat-sport population	822	83.5	954
Validated personality instrument	367	37.3	596
Genotype–personality association	282	28.6	504
Dopaminergic polymorphism analyzed	234	23.8	388
Study design	74	7.5	206
Human study population	21	2.1	54

Panel C. Number of domains rated "no" per excluded record

Domains failed	0	1	2	3	4	5–6
Records (n)	112	321	260	233	38	21

The 112 records with no domain rated "no" were excluded because they did not meet the overall screening threshold: they carried "maybe" ratings on one or more domains. The extended verification carried out during revision (Section 2.4) re-examined every excluded record rated yes or maybe on all five non-population domains whose title or abstract referred to athletes, sport or training (115 records, flagged in File S2); five proved eligible and are now included, and the remaining 110 exclusions were confirmed.
